# Digital health monitoring for adults with treatment-resistant depression: Observational feasibility study protocol

**DOI:** 10.1371/journal.pone.0333484

**Published:** 2025-10-24

**Authors:** Karisa Parkington, Reinhard Janssen Aguilar, Gyu Hee Lee, Alice Rueda, Fathima Adamsahib, Mohammad Al-Hassan, Stephanie Famuditimi, Perry Menzies, Qiaowei Lin, Wendy Lou, Sridhar Krishnan, Venkat Bhat

**Affiliations:** 1 Interventional Psychiatry Program, Department of Psychiatry, St. Michael’s Hospital, Unity Health Toronto, Toronto, Ontario, Canada; 2 Department of Biostatistics, Dalla Lana School of Public Health, University of Toronto, Toronto, Ontario, Canada; 3 Department of Electrical, Computer, and Biomedical Engineering, Toronto Metropolitan University, Toronto, Ontario, Canada; 4 Institute of Medical Science, Temerty Faculty of Medicine, University of Toronto, Toronto, Ontario, Canada; 5 Department of Psychiatry, University of Toronto, Toronto, Ontario, Canada; University of Catania, ITALY

## Abstract

Treatment-resistant depression (TRD) affects approximately 30% of adults living with major depressive disorder, resulting in many patients not achieving complete symptom remission and at risk for relapse. Digital health monitoring (DHM) using electronic data capture platforms and wearable devices is emerging as a promising therapeutic adjunct in depression. Research indicates that continuous monitoring of physical and mental health symptoms with smart devices not only helps improve patient health awareness and healthy lifestyle behaviours, but also offers scientists and clinicians insights into patients’ health patterns over time. Critically, there is a paucity of studies informing the feasibility of DHM in TRD and the efficacy of clinically meaningful digital biomarkers informing depression treatment response, remission, and relapse. Before definitive trials evaluating DHM can be designed for patients with TRD, and evidence-based recommendations can be made to inform clinical practice, pilot data is needed. The primary aim is to determine the feasibility of implementing DHM platforms to collect active (clinical assessments) and passive (wearable biometrics) health data among adult outpatients with TRD. The secondary aim is to identify and evaluate digital biomarkers of depression treatment remission and response. This single-site, observational pilot study has received research ethics board approval and recruitment is going. A DHM suite will be offered to 200 outpatients with TRD enrolled for clinical neuropsychiatric treatment. Electronic clinical assessments of anxiety (GAD-7) and depression (PHQ-9 or MADRS) will be administered on treatment days in line with standard care, and wearable devices (smart rings) will be available for participants to wear throughout the course of treatment. Descriptive and inferential group- and individual-level analyses will inform study objectives and data-driven machine learning techniques will develop personalized digital phenotype profiles of the “depressed experience” in patients with TRD. Recruitment has started and will remain open until April 2027. This pilot trial will determine the feasibility of, and provide clinical parameter estimates for, future larger trials implementing DHM platforms in TRD. This area of study might emphasize the need for personalized treatment protocols in TRD that integrate digital tools to augment the standard of care, ultimately leading to the development and advancement of more targeted and effective interventions.

**Study registration:**

Clinicaltrials.gov NCT06732089. Registered December 9^th^, 2024.

## Introduction

Major depressive disorder (MDD) affects over 300 million people worldwide and is a leading cause of disability [1]. In Canada, 1.5 million people suffer from depression every year [1], with considerable increases in prevalence rates during and post-pandemic [2,3]. Approximately 11.3% of adults will have at least one depressive episode in their lifetime [4], highlighting the impact of the mental health epidemic. However, treating MDD is challenging; despite ongoing advances in pharmacotherapy [5], one-third of patients fail to achieve symptom remission even after two suitable trials of antidepressants, leaving them inadequately treated [5,6]. These patients are considered to have treatment-resistant depression (TRD) [7,8], a condition associated with higher rates of functional impairment, economic burden, and suicidality than non-refractory depression [7–10]. These findings underscore an urgent unmet need for new treatments for MDD and, most prominently, for TRD.

Recent advancements in depression treatment have led to emerging anesthetic and neurostimulation modalities, such as repeated transcranial magnetic stimulation (rTMS) [11,12], intravenous ketamine (IVK) [13,14], and electroconvulsive therapy (ECT) [15,16], for cases where previous treatments have been unsuccessful. These emerging treatments for TRD demonstrate rapid antidepressant results, with treatment periods of 2–4 weeks. In brief, rTMS is a safe, effective, non-invasive neuromodulation therapy for MDD that directly stimulates specific areas of the brain in the prefrontal cortex using focused electromagnetic field pulses applied to the scalp [12]. These pulses stimulate specific parts of the brain and are applied repeatedly to strengthen or weaken neural connections, leading to long-lasting changes in brain activity. This change is known to reverse unhealthy and abnormal patterns of brain activity that are associated with major depression. With repeated treatments, the magnetic pulses change the neuronal activity and pathways between brain cells, returning the brain to typical functioning [12]. Another treatment option is ketamine – a general anesthetic [17] that is capable of short-term reductions in depressive symptoms and suicidal thoughts among patients with TRD when administered in sub-anesthetic doses [13]. A standard ketamine treatment is administered through 40-minute intravenous infusions twice weekly for three weeks, with rapid antidepressant effects (onset within hours of infusion) that can be sustained with repeated infusions [14,18]. Alternatively, ECT is a safe and effective treatment for several mental health conditions that have not improved from other treatments, including TRD [15,16]. In short, ECT administers a brief, controlled electrical current between two electrodes applied on the surface of the scalp or temple to stimulate a specific part of the brain. This procedure causes short (15–90 seconds), controlled seizures, thereby requiring general anesthesia [15,16]; however, ECT may be considered the most effective treatment modality in psychiatry with robust anti-suicidal effects [19].

Despite the positive outcomes of these neuropsychiatric advancements, relapse rates remain high among patients with TRD and the mechanisms delineating treatment responders from non-responders remain unclear. One possible solution to this issue may be addressed by incorporating measurement-based care (MBC) to augment traditional care [20]. Digital health monitoring (DHM), through accessory-based wearable devices and/or electronic data capture platforms, offers near real-time data capture and monitoring of mood, behaviour, and physiological parameters in naturalistic environments at a higher frequency than currently offered through synchronous standard care [21,22]. Visual feedback and interactive user platforms also allow patients to actively self-monitor their symptoms, thereby promoting healthy lifestyle behaviours [23–25]. For instance, many patients with TRD experience cognitive and memory deficits that may interfere with daily compliance [26,27]; therefore, visual feedback and app-based alerts can help patients maintain treatment adherence and compliance.

Application- and web-based platforms offer secure and convenient delivery of validated clinical scales, surveys, and ecological momentary assessments to participants. For example, the Research Electronic Data Capture (REDCap; https://www.project-redcap.org) platform offers functionalities such as defining user roles and privileges, authenticating users, encrypting data during transmission, de-identifying protected health information, and providing comprehensive auditing features to track and monitor data access and modifications. Active data collection through electronic data capture platforms offers healthcare providers insights into symptom monitoring and early detection of relevant health concerns, thereby promoting healthy behaviours and augmenting clinical care [25,28,29]. In the context of MBC, web-platforms such as REDCap are ideal for electronic data collection of clinical assessments, validated self-report questionnaires, and qualitative surveys (e.g., patient satisfaction, user experience), with flexibility for remote or in-person acquisition.

Likewise, accessory-based wearable devices (e.g., smartwatches, smart rings) can be leveraged for clinical research and practice [30–33]. More specifically, biosensors embedded in accessory-based devices enable unobtrusive, continuous recording of physiological measures of physical and mental health (e.g., activity, sleep, heart rate, body temperature, blood oxygenation) in the real world [31,32,34]. Dozens of research-grade and commercial devices (e.g., Oura Ring) are currently available on the market [35,36], with a surge in uptake and implementation in clinical research trials during and since the pandemic. Accumulating evidence demonstrates the efficacy of DHM for depression and improving patient outcomes, highlighting important options for personalized care and potential for precision medicine [30–32,37].

In light of the fluctuating nature of symptoms in TRD, electronic data capture of active (self-report) and passive (biosensor) data further permits digital phenotyping – the continuous, real-time assessment of individual human phenotypes in everyday environments using data collected from smartphones, wearables, and other digital platforms [31,38,39]. Digital phenotyping provides detailed insights into physical (e.g., activity, sleep, heart rate) and mental health symptoms (e.g., depressive symptoms, anxiety, well-being) and is central to understanding individual differences in the lived experience of mental disorders [40]. Digital phenotyping can deliver precise, longitudinal analysis of active and passive data, offering personalized monitoring of attaining treatment response and remission in patients with mental disorders in naturalistic environments in real time [41]. Treatment response is defined as a clinically meaningful reduction in symptoms relative to baseline, typically marked as symptom reduction >50% or symptom change greater than or equal to a validated scale’s standardized minimal clinically significant difference. Treatment remission is defined as symptom presentation achieving levels below the clinical threshold (e.g., score < 5 on the Patient Health Questionnaire, 9-items; PHQ-9).

More specifically, passive data is effective at identifying behaviours and trends in activity but is poor in measuring people’s internal states, motivation, and attitude, whereas active data is the opposite. Developing a methodology for integrating the two data sources can mitigate their respective weaknesses. These advancements have the ultimate potential for informing the development and implementation of symptom alert detection and prediction applications (apps) for early diagnosis and intervention. Thus, DHM and digital phenotyping in patients with TRD is necessary to identify and classify clinically useful markers that can be used to refine diagnostic processes, tailor treatment choices, and improve condition monitoring [31,38,39,41,42], with significant implications for patient care and the field of digital psychiatry.

There are concerns that the use of digital platforms may affect the therapeutic relationship, and while barriers are apparent (e.g., patients’ feelings of isolation, therapists’ concerns of violating therapeutic boundaries) [43], no harmful effects have been reported. Instead, many positive benefits have been described, including improved communication, interaction and clinical discussion; clinicians’ feelings of compassion and empathy; and patients’ feelings of reassurance, comfort, connection, trust and accountability [43,44]. Emerging studies demonstrate that implementation of digital mental health platforms leads to comparable or enhanced quality of the patient-therapist alliance, relative to standard care [45]. App engagement and acceptability have also been shown to depend on the therapeutic relationship [46–48]. Collectively, the state of the literature recommends incorporating DHM platforms as adjuncts to standard care to support patient engagement and treatment adherence [45]. Two other systematic reviews on mental health apps reported that dropout rates tended to be lower in studies offering human feedback and interventions such as in-app self-monitoring [49,50].

Given the rapid-acting antidepressant effects and high relapse rates for neuropsychiatric treatment in TRD, there is a critical need to examine the feasibility of multimodal DHM to enhance symptom monitoring of adults with TRD receiving neuropsychiatric treatment and move towards personalized care. However, there is a notable gap in studies implementing DHM in patients with TRD. To address this, we propose the concurrent delivery of DHM in tandem with TRD patients’ neuropsychiatric (rTMS, IVK, or ECT) treatment.

The Interventional Psychiatry Program (IPP) at St. Michael’s Hospital specializes in the delivery of anaesthetic and neurostimulation treatments for depression [51]; the treatment algorithm for TRD is illustrated and described in S1 Fig. At the forefront of psychiatric treatment innovation, the IPP leads clinical trials dedicated to monitoring and reporting rates of mental health issues (e.g., anxiety, distress, depression, self-harm and suicide) to understand mechanisms and inform intervention, in line with key global mental health priorities [52]. In particular, the Digital Interventions and Intelligence Group (DiiG) [53] focuses on the development, implementation, and evaluation of digital platforms to improve the diagnosis and treatment of mood disorders.

### Objectives

The primary objective of this study is to determine the feasibility of DHM (using web-based clinical assessments and wearable devices) among adult outpatients with TRD. Secondary aims will inform the relationships between self-reported symptoms and objective physiological measures over the course of neuropsychiatric treatment (rTMS, IVK, or ECT), to index treatment response and remission in TRD. Exploratory data-driven approaches will also seek to create personalized digital phenotype profiles of the depressed experience based on digitally acquired data.

## Methods

All informed consent procedures and study activities were approved by the Unity Health Toronto Human Research Ethics Board at St. Michael’s Hospital (Project No. 21–274; Protocol v3.2) on October 8^th^, 2024 and registered on ClincalTrials.gov (NCT06732089) on December 9^th^, 2024. All research activities will be conducted in accordance with the ethical principles laid down in the Declaration of Helsinki, the protocol (S2 File Protocol), Good Clinical Practice guidelines, and applicable privacy legislation, including the Health Insurance Portability and Accountability Act (HIPAA) and Personal Information Protection and Electronic Documents Act (PIPEDA).

### Study design

**Fig 1:**
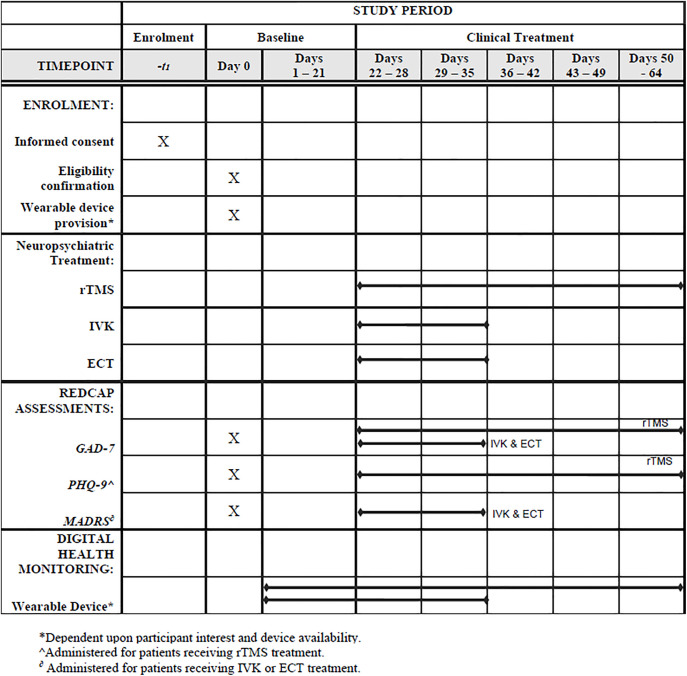
Standard protocol items – Recommendations for interventional trials (SPIRIT) schedule. Overview of study enrolment, study procedures, and timeline for adults with TRD participating in this observational study.

This observational pilot study will assess the feasibility of implementing DHM in tandem with a single course of neuropsychatric clinical care (rTMS, IVK, or ECT) for TRD patients. Specifically, a web-based data capture platform (REDCap; https://www.project-redcap/org) and wearable devices (smart rings) will be provided in parallel with treatment as usual (TAU) in the IPP (Fig 1 and S3 Checklist). Recruitment started on January 6^th^, 2025 and will remain open for approximately two years, ending by December 15^th^, 2026. Participants will be enrolled in the study for the duration of their clinical treatment in the IPP (approximately 2–4 weeks) and, if necessary, a 2–3-week baseline period (prior to starting treatment) to establish baseline parameters for predictive algorithms of wearable devices.

Following enrollment to the IPP and provision of informed written consent (S4 File), data from clinical assessments completed as part of clinical care (Generalized Anxiety Disorder – 7 item Scale (GAD-7) [54] and either the PHQ-9 [55] or Montgomery-Åsberg Depression Rating Scale (MADRS) – depending on the treatment arm) will be entered into REDCap after each treatment session (Fig 1). Wearable devices (smart rings) will also be available for participants to wear throughout the duration of treatment, if interested, to be returned on the last treatment day. The smart ring used for this study (Oura Ring) requires a pre-treatment baseline period (2–3 weeks) to establish reliable recordings and personalized features for the underlying predictive algorithms driving data visualization and scores in the associated smartphone app. Thus, participants will receive their wearable device approximately two weeks before starting treatment. Participants who opt not to use a device can begin treatment immediately upon enrollment.

### Sample size estimates

This observational study will target a sample size of 200 participants with TRD, consistent with digital phenotyping study recommendations [56] and treatment response goals. This large sample size will allow for adequate distribution of heterogenous sub-samples across three treatment arms, high power (>80%) to detect symptom changes, and provides flexibility for potential dropouts (maximum 20% (n = 40) to be deemed feasible) and engagement metrics.

### Participant eligibility

This study will include adults (aged 18+) enrolled in the IPP at St. Michael’s Hospital diagnosed with MDD (without psychotic symptoms) [57], capable of providing informed consent. Participants’ cognitive and linguistic capacity to provide informed consent was determined through researcher observations through a structured eligibility screening and consent discussion process. Participants must be currently experiencing a major depressive episode (i.e., MADRS ≥ 20) and be considered treatment-resistant (i.e., failed response to two or more adequate antidepressant trials during the current episode). Study participation will not alter the standard of care, including any required changes to treatment regimens. Participants may continue to receive concurrent psychotherapy and/or pharmacological treatments as part of their usual care throughout the study. However, to remain eligible for the observational study, no treatment changes, aside from those received through the IPP, are allowed one month (28 days) before enrollment, or during the entire duration of treatment due to the probable impact on brain activity and psychiatric symptoms. Medications will be monitored on a regular basis and may be restricted as part of the standard of care for their respective clinical treatment. If any changes to concomitant psychotropic medications are required as per the patient’s standard of care, the participant will be withdrawn from the study.

To be eligible to receive a wearable device, participants must own a smartphone with Bluetooth capabilities. Regular Internet access (e.g., Wifi, data plans) will be required to access and sync data with the digital platforms and English fluency will be required to complete all study activities independently.

### Procedures

#### Recruitment.

Patients who have been referred to the IPP will undergo a comprehensive intake with a psychiatrist during their first visit. This clinical evaluation will be used to determine a specific clinical care plan for each patient based on the patient’s psychiatric history, current presentation, treatment indications, past treatment response, and patient preferences. Treatment options may include rTMS, IVK or ECT. This observational study will follow TRD patients through a single course of neuropsychiatric treatment (rTMS, IVK, or ECT).

**Fig 2:**
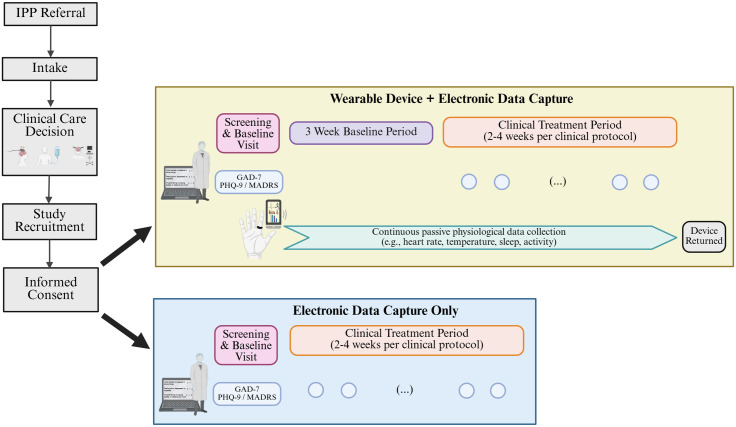
Study design.

An individual from the program’s clinical team will inform newly referred patient-participants that digital platforms are available to facilitate the collection of physical and mental health data. If patients are interested in participating, they will be directed to a member of the research team, who will provide more information about the study and obtain informed consent. Upon receiving informed consent, participants will be assigned de-identified study identifiers and members of the clinical team will enter relevant demographic and medical history information (obtained during IPP intake) into the REDCap platform (Fig 2).

#### Screening and baseline visit.

For participants opting to use a wearable device (smart ring), the first visit (i.e., Screening and Baseline Visit) will be scheduled approximately 2–3 weeks before the first treatment session. For participants opting to only include REDCap data, the Screening and Baseline Visit will correspond to the first clinical treatment appointment (Fig 2). Here, the clinical team will review participants’ demographic information and medical history and collect baseline anxiety (GAD-7) and depression (PHQ-9 or MADRS) symptoms. Participants receiving a wearable device will also be fitted with an appropriately sized ring and the Oura app will be set up on participants’ smartphones using their de-identified study credentials. The clinical team will briefly introduce participants to the app platform; technical assistance will be supported by the research team.

#### Electronic clinical assessments.

Case report forms (demographics, medical history form), self-report questionnaires (GAD-7 and PHQ-9), and clinician-administered assessments (MADRS) will be completed using the REDCap platform (https://www.project-redcap.org). This open-source, web-based clinical data management and electronic data capture system and database is supported by the Applied Health Research Centre at Unity Health Toronto, in compliance with institutional privacy guidelines, the HIPAA [58], PIPEDA [59], and Food and Drug Administration regulations [60]. Each treatment day, as part of their clinical care, participants will complete the self-report questionnaire(s) and clinical assessment relevant to their standard of care protocol. In the IPP, all patients complete the GAD-7 as part of routine care. Patients receiving rTMS treatment complete the PHQ-9 as the clinical measure of depression; patients receiving IVK or ECT treatments complete the MADRS with their clinical care team. Responses to these assessments will be entered onto REDCap by the clinical research team after each treatment session.

#### Wearable devices.

Commercial smart rings (Oura Ring) will be available for participants with a compatible smartphone. The Oura Ring requires a 2–3-week baseline acquisition period to improve personalization features. Participants will be asked to wear their smart ring daily throughout the study, recharging it when necessary (~20 minutes every 3–4 days). To synchronize passively collected data with the smartphone app and the Application Programming Interface (API) server, participants will be instructed to open the Oura app daily while connected via Bluetooth and Wi-Fi. A member of the research team will monitor data completion and notify the clinical team if any participants are missing wearable data, so that reminders can be provided at the next treatment session and reasons for data loss can be explored (e.g., technical issues, participant wanted a break, forgot to charge or put on the device). Participants will also be encouraged to view their physical and mental health parameters in the smartphone app (e.g., time-series graphs; predictive scores of activity, sleep, and readiness). Wearable devices will be returned at the end of the last treatment session.

#### Clinical treatment for TRD.

Participants will receive the standard protocol for one of the clinical treatments offered at the IPP for TRD (rTMS, IVK, or ECT). During clinical visits, the team will review patients’ self-reported scores of anxiety (GAD-7) and depression (PHQ-9 or MADRS) on REDCap.

### Data security

All digital platforms used in this study are protected by multiple levels of authentication and authorization adhering to patient privacy laws (e.g., HIPAA, PIPEDA) and have been approved by the Unity Health Toronto Privacy and Security Office. Passive data collected from the wearable devices will be made accessible through their respective APIs. Importantly, the wearable device apps and servers are independent, and stored separately, from the REDCap platform. Therefore, data are neither linked across platforms nor related to any other platform except through the de-identified credentials assigned to the participant. No personal information will be collected through the digital platforms and only authorized members of the research team will have access to the data. Each participant’s de-identified data will be exported and stored on an external encrypted and password-protected hard-drive and the data will only be associated with a participant identification number. Electronic data will be stored for a minimum of seven years and may be used for other research or analyses by the investigators, or by other researchers.

Furthermore, a two-zone approach will be implemented by designated study team members to one of two groups: identified or de-identified. As the names suggest, the identified group (e.g., research coordinator, clinical team) will have access to identified (i.e., demographic data) *and* de-identified information and will thus be able to re-identify the data to provide data management, quality, and data linkage functions. Alternatively, the de-identified group (e.g., data analysts) will only have access to the de-identified information. This will ensure that certain team members only have access to de-identified data and will not have access to any of the participant’s identifiers to protect participant privacy.

### Data analysis plan

#### Feasibility analysis.

DHM implementation feasibility will be detailed using descriptive statistics. Categorical data (e.g., demographics, medical history) will be represented with frequencies (counts and proportions); continuous data (e.g., age, baseline depression scores) will be informed by means and standard deviations (for parametric, normally distributed data) or median and interquartile range (IQR; for non-parametric, skewed data distributions). To quantify engagement and tolerability, the proportion lost to follow-up will be estimated along with a 95% confidence interval (CI); for DHM to be deemed feasible, the upper 95% confidence limit should not exceed 20%. The proportion compliant with the protocol, including treatment compliance as well as study completion and complete data on clinical outcomes (i.e., the per-protocol group) will be estimated with a 95% CI. For validity, this should be fairly high so that the lower 95% confidence limit is > 80%. Reasons for data loss, poor engagement, and participation withdrawal will be explored to inform patient feasibility and user experience.

#### Statistical analysis.

To inform preliminary estimates of DHM sensitivity and efficacy for detecting clinically relevant changes in physical and mental health symptoms, pre-post treatment effects will be analyzed using group-level biostatistics (paired *t-*tests and analyses of covariance for parametric data; Wilcoxon signed-rank test for non-parametric data). Age and sex will be considered as covariates; the potential impact of other demographic and medical history factors (e.g., ethnicity, religious affiliation, family history of depression) on treatment response will also be explored. Within- and between-person correlations of subjective and objective mental health measures will be conducted to apprise individual differences and variability. This will be further supplemented by regression, multilevel modeling, and advanced data analytic techniques to inform individual-level trends and digital features predictive of treatment outcomes (response and remission).

#### Machine learning and digital phenotyping.

Machine learning and advanced analytics (e.g., dimensionality reduction, statistical relevance or multi-view biclustering) will be used to fuse passive (physiological) data with active (clinical assessments) data. This approach will permit the cross-validation and improvement of measurements, the explanation of human behaviour, and novel opportunities to improve causal inference in experimental settings. The active (mental health symptom) data will serve as a ground truth to validate the passive (physiological) data for monitoring a “depressed experience”. To identify and predict symptoms of distress and depression, we will use generalized machine learning models, followed by the development of personalized models tailored to each participant’s unique longitudinal data. These models will be built using individual-level patterns in self-reported symptoms and wearable data, allowing us to generate participant-specific predictions. Because this is an iterative process, models will be refined as more data are collected. To combat missing data, we will apply filtering techniques and use algorithms estimate and interpolate gaps, ensuring analyses remain robust and reliable.

**Fig 3:**
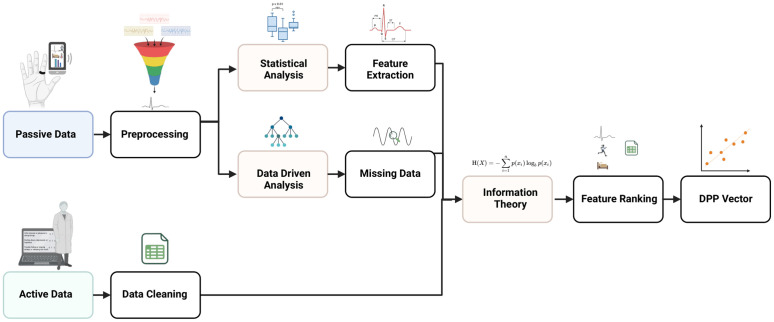
Block diagram representation of the proposed SID pipeline.

Digital biomarker features (e.g., heart rate variability, sleep staging, activity levels) will be extracted from the physiological data for better representation using machine learning trends, classifications and relationships. Signal processing will pre-process physiological recordings to remove poor signal quality, noise, and artifacts. Our goal is to create a systematic and individualized representation of each participant’s data (i.e., digital phenotype profile, DPP) by combining multiple types of information, including self-reported symptoms (active data) and physiological signals from wearable devices (passive data). To achieve this, we use a Statistics, Information Theory, and Data-driven (SID) pipeline (Fig 3) that establishes a personalized baseline for each participant and continuously monitors for meaningful changes over time. This is accomplished using a mathematical technique known as robust principal component analysis (RPCA), which helps identify key patterns and variations by simplifying the data into concise and interpretable structure.

The resulting DPP serves as a summary of a participant’s physical and behavioural health, drawing from both self-report and wearable data. As new data are collected over time, the models are continuously fine-tuned to reflect individual changes. While this approach uses advanced techniques like RPCA, further validation is needed to ensure these models remain accurate and reliable across diverse participants. To enhance personalization, we will generate a refined version of the DPP, the personalized digital phenotype profile (pDPP), which uses longitudinal data to track how an individual’s health patterns evolve over time. These profiles are built by integrating each participant’s physiological data with their self-reported mental health symptoms. Once established, the model can begin to infer aspects of a participant’s mental well-being using only passive physiological data, reducing the burden of frequent self-reporting. Over time, these personalized models may offer a non-intrusive way to support real-time monitoring and improve mental healthcare, especially in individuals with TRD.

## Discussion

Digital health monitoring is emerging as a potential adjunctive for TRD [31,38]. Here, we have outlined the protocol for an observational pilot trial that will deliver DHM in tandem with neuropsychiatric treatment for TRD (rTMS, IVK, or ECT). Wearable devices (Oura Ring) and electronic data capture of anxiety (GAD-7) and depression (PHQ-9 or MADRS) assessments will be incorporated alongside standard of care. The primary objective of this study is to assess feasibility and acceptability of digital health monitoring in TRD, with secondary objectives targeting preliminary insights into efficacy and sensitivity of these digital platforms for capturing clinically meaningful changes in treatment response and remission over the course of treatment. Exploratory aims will explore data-driven insights from machine learning and digital phenotyping methods, with the goal of developing pDPPs for patients with TRD.

While previous studies have demonstrated the suitability of MBC and DHM with wearable devices in depression [31,34–36,38], there is a need for ongoing research to identify reliable digital biomarkers of treatment response and remission in TRD. This will be the first study to deliver DHM in tandem with neuropsychiatric treatment for TRD to inform future clinical trials and psychiatric care protocols. Digital data capture will allow our team to determine participants’ mental health trajectories using both active (self-report and clinician-administered) and passive (device-based) measures, assess the relationship between treatment response/remission and digital mental health phenotypes, and track stability of changes in outcomes over time. By offering patients’ access to wearable devices with real-time health monitoring and predictive modeling capabilities, remote MBC may promote self-monitoring of systems and promote healthy behaviours and lifestyle changes, which in turn can contribute to improvements in mental health [24,25]. Self-monitoring of physical and mental health symptoms has implications for health awareness and can affect patients’ perceptions of their progress. Supervision and delivery of digital mental health programs by trained personnel have also been shown to predict lower dropout rates in psychiatric samples [61].

Despite this pilot study’s innovative contributions to the literature on DHM for TRD, some limitations should be noted. The observational nature of this study will result in unequal numbers of participants across treatment arms (non-random assignment). While this study design is appropriate for the purpose of feasibility evaluation, it will limit the types of statistical analyses that can be performed and interpretability of secondary outcome comparisons (clinical efficacy), thereby precluding a definitive understanding of the relationships between digital data patterns and clinical outcomes across treatment arms. Moreover, the absence of post-intervention follow-up prevents sustainability analyses and definitive long-term symptom changes (maintenance or relapse). To address these disparities, quasi-experimental (e.g., matched cohort) and randomized controlled trial designs with longitudinal follow-up periods (6 months to 2+ years) and definitive sample sizes across treatment arms will be necessary. Furthermore, mixed methods approach combining qualitative insights through user experience surveys and/or semi-structured interviews should also be considered to provide a deeper understanding of patient experiences and DHM adherence. Data obtained through this observational study will determine the feasibility of, and provide clinical parameter estimates for, future larger trials implementing DHM in TRD. This line of research might emphasize the need for digital health monitoring in outpatient psychiatric care and TRD clinical trials and could lead to the development of more targeted and effective interventions, thus improving outcomes of TRD, including quality of life.

## Supporting information

S1 Fig Overview of the clinical treatment algorithm for adult patients with TRD within the IPP. Referred TRD patients first undergo TMS. Once a clinically meaningful treatment response or remission is achieved after at least 20 rTMS sessions, patients are discharged from the IPP. In the case of persistent failure to achieve clinical remission, and provided there are no contraindications, patients then undergo IVK treatment (4 sessions) and, if necessary, ECT. This observational study will incorporate a DHM suite (electronic data capture and wearable devices) in tandem with neuropsychiatric standard of care (rTMS, IVK, or ECT). Figure created in BioRender. Bhat, V. (2025) https://BioRender.com/c61t762.
(TIF)

S2 File Protocol v3.2. (PDF)

S3 Checklist SPIRIT checklist. (DOC)

S4 File Informed consent form v3.2. (PDF)
