# Supplementary figures and images for: Digital health monitoring for adults with treatment-resistant depression: Observational feasibility study protocol
Source: PLoS One. 2025 Oct 24;20(10):e0333484. doi: 10.1371/journal.pone.0333484 (PMC12551856; doi:10.1371/journal.pone.0333484)

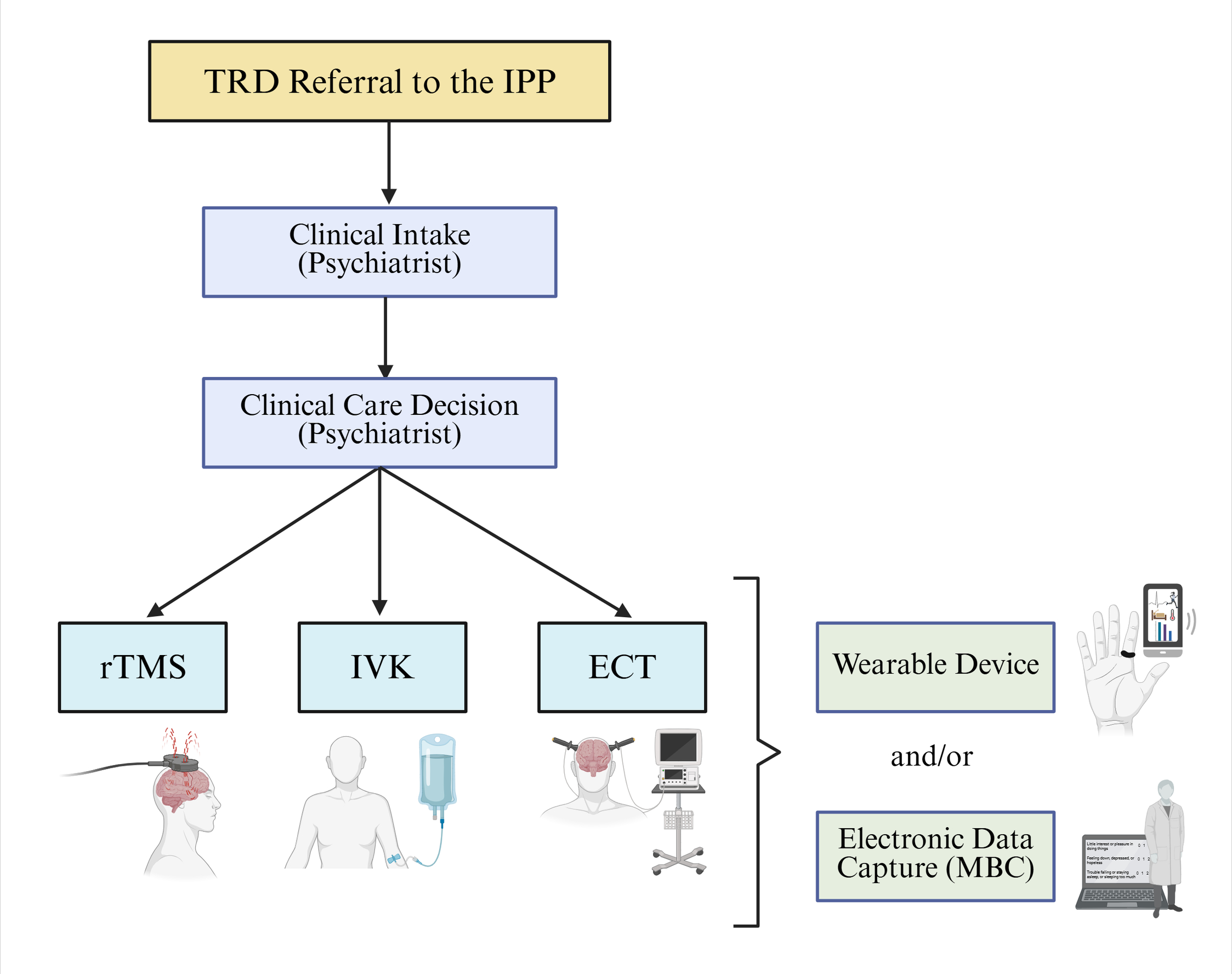

Supplement: S1 Fig — Referred TRD patients first undergo TMS. Once a clinically meaningful treatment response or remission is achieved after at least 20 rTMS sessions, patients are discharged from the IPP. In the case of persistent failure to achieve clinical remission, and provided there are no contraindications, patients then undergo IVK treatment (4 sessions) and, if necessary, ECT. This observational study will incorporate a DHM suite (electronic data capture and wearable devices) in tandem with neuropsychiatric standard of care (rTMS, IVK, or ECT). Figure created in BioRender. Bhat, V. (2025) https://BioRender.com/c61t762. (TIF) [file pone.0333484.s001.tif]
