# Supplementary material for: Digital health monitoring for adults with treatment-resistant depression: Observational feasibility study protocol
Source: PLoS One. 2025 Oct 24;20(10):e0333484. doi: 10.1371/journal.pone.0333484 (PMC12551856; doi:10.1371/journal.pone.0333484)
Supplement: S2 File — (PDF) [file pone.0333484.s002.pdf]

Determining the Feasibility of Digital Interventions for Adults with Treatment-Resistant Depression

Version 3.2 – 16 September 2024

Investigational Products	Digital Interventions: Web-based platform (REDCap) and wearable device (Oura Ring)
Indication	Adults suffering from treatment-resistant depression who are being treated in the Interventional Psychiatry Program
REB #	21-274
Study Principal Investigator:	**Venkat Bhat**, MD MSc FRCPC<br>St. Michael's Hospital and the University of Toronto
Study Co-Investigators:	**Perry Menzies**, MD MSc FRCPC<br>St. Michael's Hospital and the University of Toronto<br><br>**Wendy Lou**, PhD<br>Dalla Lana School of Public Health, University of Toronto<br><br>**Sri Krishnan**, PhD PEng FCAE<br>Signal Analysis Research Lab and Toronto Metropolitan University
Study Coordinator:	**Karisa Parkington**, PhD<br>St. Michael's Hospital, Unity Health Toronto
Study Staff:	**Fathima Adamsahib**, BSc, PA-C<br>St. Michael's Hospital, Unity Health Toronto<br><br>**Reinhard Janssen Aguilar**, MA, MD<br>St. Michael's Hospital, Unity Health Toronto<br><br>**Gyu Hee (Sarah) Lee**, BSc<br>St. Michael's Hospital, Unity Health Toronto
Funder:	Miner's Lamp Innovation Fund in Prevention and Early Detection of Severe Mental Illness by the Department of Psychiatry at University of Toronto

Abbreviations

API	Application Programming Interface
ATHF	Antidepressant Treatment History Form
CRF	Case Report Form
C-SSRS	Columbia-Suicide Severity Rating Scale
DiiG	Digital Intelligence and Interventions Group
DPP	Digital Phenotype Profile
ECG	Electrocardiogram
ECT	Electroconvulsive Therapy
EEG	Electroencephalography
EMG	Electromyography
EMA	Ecological Momentary Assessment
GAD-7	Generalized Anxiety Disorder scale
GPS	Global Positioning System
GSR	Galvanic Skin Response
HRV	Heart Rate Variability
IPP	Interventional Psychiatry Program
ICT	Information and Communication Technologies
IVK	Intravenous Ketamine
MADRS	Montgomery-Asberg Depression Rating Scale
MDD	Major Depressive Disorder
MDE	Major Depressive Episode
ML	Machine Learning
MINI	Mini International Neuropsychiatric Interview
pDPP	Personalized Digital Phenotype Profiles
PHIPA	Personal Health Information Protection Act
PIPEDA	Personal Information Protection and Electronic Documents Act
PHQ-9	Patient Health Questionnaire
PPG	Photoplethysmography
RAM	Random Access Memory

REB	Research Ethics Board
REDCap	Research Electronic Data Capture
RESP	Respiration
RHR	Resting Heart Rate
RSA	Rivest–Shamir–Adleman algorithm
rTMS	Repetitive Transcranial Magnetic Stimulation
RPCA	Robust Principal Component Analysis
SSD	Solid State Drive
SMH	St. Michael’s Hospital
SID	Statistics, Information Theory and Data-driven
TRD	Treatment-Resistant Depression
TLS	Transport Layer Security protocol
UHT	Unity Health Toronto
VM	Virtual Machine
WHO-5	5-item World Health Organization Well-Being Index

Table of Contents

Abbreviations....2
Table of Contents....4
1. TRIAL SUMMARY....6
2. INTRODUCTION....8
    2.1. Background and Rationale....8
    2.2. Objectives....12
        2.2.1 Personal Digital Phenotype Profile Definition....13
3. METHODS....14
    3.1. Study Design....14
    3.2. Eligibility Criteria....16
        3.2.1. Inclusion Criteria....16
        3.2.2. Exclusion Criteria....16
    3.3. Withdrawal Criteria....17
    3.4. Concomitant Medications....17
    3.5. Concomitant Care....18
    3.6. Interventions....18
        3.6.1. REDCap....18
        3.6.2. Oura Ring Wearable Device....18
    3.7. Outcomes....20
    3.8. Procedures....22
        3.8.1. Study Schedule....22
        3.8.2. Recruitment....23
        3.8.3 Informed Consent....24
        3.8.4 Screening and Baseline Visit (~1 Hour)....24
        3.8.5 Intervention....26
            3.8.5.1 Standard of Care....26
            3.8.5.2. Digital Intervention Data Collection....26
                3.8.5.2.1. Active Data – Assessments via REDCap....26
                3.8.5.2.2. Passive data through Oura Ring....27
            3.8.5.3 Demographic Data and Medical History....29
        3.8.6 Follow-up....30
    3.9. Reimbursement....30
    3.10. Quality Assurance....30
4. DATA MANAGEMENT....30
    4.1. Security and Privacy of the Study Data....31

4.1.1. Oura Ring Wearable Device ... 31
4.1.3. REDCap ... 32
5. DATA ANALYSIS METHODS ... 33
5.1 Statistical Analysis ... 33
5.2. Personal Digital Phenotype Analysis and Generation ... 34
5.3. Interpretation of the Personal Digital Phenotype Profile ... 35
5.4. Sample Size Considerations ... 35
5.5. Qualitative Analysis ... 36
6. ETHICS AND DISSEMINATION ... 36
6.1. Research Ethics Considerations ... 36
6.2. Protocol Amendments ... 36
6.3. Informed Consent Process ... 36
6.4. Exclusion of Women, Minorities, and Children (Special Populations) ... 37
6.5. Confidentiality ... 37
6.6. Study Discontinuation ... 37
6.7. Declaration of Interests ... 38
6.8. Dissemination Policy ... 38
6.9. Authorship ... 38
7. IMMEDIACY & IMPORTANCE OF RESEARCH IMPACT ... 38
8. STUDY ADMINISTRATION ... 38
8.1. Key Contacts ... 38
8.2. Funding ... 39
8.3. Roles and Responsibilities ... 39
8.3.1. Sponsor and Funding ... 40
9. REFERENCES ... 40
10. APPENDICES ... 43
Appendix A. Questionnaires ... 43
Appendix B. CRF – Demographics ... 43
Appendix C. CRF-Eligibility ... 43
Appendix D. CRF- Medical History Form ... 43
Appendix E. Exit Interview ... 43
Appendix F. Email Drafts ... 43
Appendix G. CRF – Antidepressant Treatment History Form ... 43
Appendix H. Screening+Baseline Visit Script ... 43

1. TRIAL SUMMARY

Title	Determining the Feasibility of Digital Interventions for Adults with Treatment-Resistant Depression
Principal Investigators	Venkat Bhat, MD MSc FRCPC<br>St. Michael's Hospital and the University of Toronto<br>venkat.bhat@utoronto.ca<br>(416) 360-4000 x76404
Brief title	Digital Interventions for Adults with Treatment-Resistant Depression:<br>A Feasibility Study
Condition(s) or focus of study	Patients with treatment-resistant major depressive disorder (MDD) undergoing treatment within the Interventional Psychiatry Program at St. Michael's Hospital
Key eligibility criteria	Inclusion Criteria:<br>1. Adults 18+ who are capable of giving informed consent<br>2. Patients meeting diagnostic criteria for major depressive disorder (MDD) without psychotic symptoms according to the Diagnostic and Statistical Manual of Mental Disorder – Fifth Edition (DSM-5)[1] and currently experiencing a Major Depressive Episode (MDE) as confirmed at intake<br>3. A Montgomery-Asberg Depression Rating Scale (MADRS)[3] score $\geq$ 20 at screening (moderate-to-severe MDE)<br>4. Patients meeting criteria for treatment-resistant depression (TRD; failure of two or more adequate trials of antidepressant therapy of adequate dose and duration during the current episode) as confirmed at intake<br>5. Enrollment in one of the treatment modalities at the Interventional Psychiatry Program at St. Michael's Hospital, Unity Health Toronto<br>6. Ownership of a smartphone (for participants using the Oura Ring)<br>Exclusion Criteria:<br>1. Subjects without internet access, as an internet connection is required for using the study platforms<br>2. Medication changes, aside from the treatment received through the IPP, one month (28 days) before screening, or during the entire duration of treatment (variable based on treatment arm).

	3. Other exclusion criteria for this study will follow those from the clinical treatment the participant is receiving (standard of care)<br>Non-English-speaking individuals are excluded because the ability to communicate study information, answer questions accurately and completely about the study, and obtain consent are necessary.
Study design	Feasibility pilot study with clinical observation and retrospective data analysis
Date of enrollment	Start date: Dependent upon REB Approval<br><br>End date: Dependent upon REB approval
Target sample size	Target number: 200<br>No recruitment limit
Primary outcome	To determine the feasibility of using a web-based digital platform (REDCap) and commercially available wearable device (Oura Ring) to collect active and passive data, respectively, among adults receiving clinical treatment at the IPP for TRD.
Secondary outcomes	1. To evaluate the association of active data from REDCap and passive data from Oura Ring over the duration of clinical treatment with the treatment outcomes of response (reduction of $\geq$ 50%), remission (score $\leq$ clinical threshold), and relapse (score $\geq$ clinical threshold after initial remission).<br>2. To create personalized digital phenotype profiles (pDPPs) based on wearable and web-based data.

2. INTRODUCTION

2.1. Background and Rationale

Major depressive disorder (MDD) affects over 300 million people and is a leading cause of disability worldwide.[5] In Canada, 1.5 million people suffer from depression every year,[5] and 11.3% will have at least one depressive episode in their lifetime.[6] Treating MDD is a challenge-despite continuous progress in pharmacotherapy,[7] a third of patients do not achieve symptom remission with at least two adequate trials of antidepressants and remain inadequately treated[.7,8] These patients are considered to have treatment-resistant depression (TRD),[9] a condition associated with higher rates of functional impairment, economic burden and suicidality than non-refractory depression.[10,11] These findings underscore an urgent unmet need for new treatments for MDD and most prominently, for TRD.

In the Interventional Psychiatry Program (IPP)[12] at St. Michael's Hospital (SMH), we offer novel interventional and psychopharmacological treatments for depression and other comorbid disorders where previous treatments have been unsuccessful. These interventions include intravenous ketamine (IVK),[13] repeated transcranial magnetic stimulation (rTMS),[14] electroconvulsive therapy (ECT),[15] and other emerging anesthetic and neurostimulation modalities.

A key global mental health priority is to monitor and report rates of mental health issues such as anxiety, distress, depression, self-harm and suicide to understand mechanisms and inform intervention.[16] To address this priority, the IPP has developed another research stream, which focuses on the use of digital platforms to improve the diagnosis and treatment of mood disorders.[17] By using digital platforms in mental health, we can perform digital phenotyping, which is defined as the "moment-by-moment quantification of the individual-level human phenotype *in situ* using data from smartphones and other personal digital devices".[18] Given the temporally dynamic nature of symptoms within mental disorders, digital phenotyping may offer a fine-grained comprehension of mental health symptoms (e.g., mood and anxiety symptoms), motor behaviors and physiological parameters (e.g., heart rate and sleep patterns) and their

relations to environmental stimuli by quantifying the individual-level experience of illness in naturalistic settings.[19,20]

Digital platforms are central to understanding the "distress experience", which can be mapped from moment-to-moment. Digital phenotyping can accurately provide longitudinal information provision, and may offer a window of opportunity for personalized monitoring of sustained treatment response and relapse prediction in patients with mental disorders in naturalistic environments in real time.[21] Digital platforms can collect ecological momentary assessments (EMAs), offering the opportunity for self-management of mental well-being and early intervention, as well as promote healthy behaviors and augment clinical care.[22-24] In addition, digital platforms, such as wearable devices can continuously collect data unobtrusively to obtain insights on the state of mental health and well-being such as activity levels, sleep patterns and physiological parameters.[25,26] These benefits are significant to the field of psychiatry, particularly for objective measurements of mental health symptoms and to develop clinically useful markers that can be used to refine diagnostic processes, tailor treatment choices, and improve condition monitoring.[27] However, while there is great potential for using digital platforms in psychiatry, there is a critical gap in systematic studies that examine the utility of digital interventions for monitoring patients with TRD. In this study, we will evaluate the feasibility of collecting real-time passive and active data in adult patients receiving clinical treatment for TRD at the IPP. In addition, we will explore the association between the active and passive data collected and the potential ability of these real-time data to predict clinical outcomes (i.e., response, remission, and relapse) in patients with TRD. Furthermore, we will create a personal digital phenotype profile (pDPP) based on wearable and web-based data.

This project develops an evidence-base framework using digital platforms to longitudinally monitor the mental health of patients receiving treatments at the IPP for TRD. Treatments include IVK, rTMS, and ECT.[12] The treatment algorithm for TRD at the IPP is illustrated in Figure 1. Please note that only neuropsychiatric treatments offered in the IPP (rTMS, IVK, and ECT) are included in this study protocol as treatments for TRD.

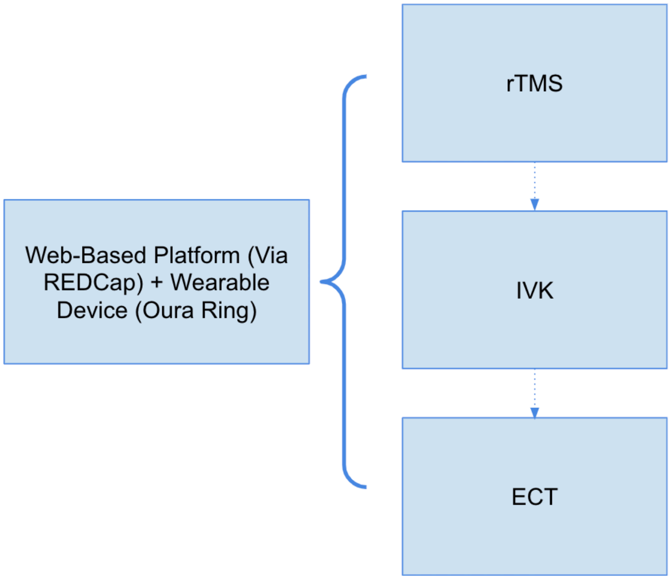

Figure 1 - Overview of current clinical research arms for TRD within the Interventional Psychiatry Program. Consenting patients with TRD referred to the IPP will first undergo rTMS. In case of persistent failure to achieve clinical remission, and provided there are no contraindications, patients then undergo IVK treatment and, if necessary, ECT. This study will incorporate a digital mental health suite (mobile assessments and wearable devices) in addition to the treatment-as-usual process in the IPP.

In the Interventional Psychiatry Program (IPP), rTMS is the first treatment arm for patients with TRD. rTMS is a safe, effective, non-invasive neuromodulation therapy for MDD based on the use of magnetic fields to modulate neural circuitry involved in emotion regulation and depressive symptoms.[28] Specifically, it works by directly stimulating specific areas of the brain using focused electromagnetic field pulses that are applied using a magnetic coil placed against the scalp, over the prefrontal cortex.[28] These pulses stimulate specific parts of the brain and are applied repeatedly to strengthen or weaken connections within the brain, leading to long-lasting changes in brain activity. This change is known to reverse unhealthy and abnormal patterns of brain activity that are associated with major depression. With repeated treatments, the magnetic pulses change the activity of the brain cells (neurons) and the pathways between brain cells, returning the brain to normal functioning.[28]

Intravenous ketamine is the second intervention arm in our treatment algorithm. Ketamine is a general anesthetic with analgesic properties approved more than 50 years ago[29] and is on the World Health Organization's list of Essential Medications as an anesthetic.[13] Over the last 20 years, ketamine has been examined in sub-anesthetic doses to treat psychiatric disorders,

including individuals who have not responded well to conventional treatments. Several short-term studies have shown that ketamine is able to reduce depressive symptoms and suicidal thoughts among patients with TRD.[13] A standard ketamine treatment is administered through IV infusions 2 times per week for 3 weeks, and every infusion lasts for about 40 minutes. Additionally, ketamine's antidepressant effects have a rapid onset (within hours) and could be sustained after repeated infusions.[30]

The last treatment arm in the IPP comprises ECT, which is reserved for patients who fail to respond to rTMS and IVK. ECT is a safe and effective treatment for several mental health conditions, including depression, bipolar disorder, and schizophrenia, which have not improved from other treatments.[15] Also, it may be considered the most effective treatment modality in psychiatry and with robust anti-suicidal effects.[31] ECT involves administering a brief, controlled electrical current between two electrodes applied on the surface of the scalp or temple. The current stimulates a specific part of the brain and causes a small, controlled seizure which lasts between 15 and 90 seconds, and the whole procedure is performed under general anesthesia.[15]

In this study, we will be assessing the uptake and feasibility of using a web-based platform (Research Electronic Data Capture – REDCap) and a wearable device (Oura Ring) to collect data from adults receiving treatment for TRD, using the following metrics: number of recruitments, recruitment rates, dropout rates and reasons for dropping out, and data completion rates. In addition, by collecting dynamic digital (active and passive) data, we will be able to analyze this data with respect to treatment response, remission, and relapse rates for each treatment arm. Treatment response is defined as a 50% or greater decrease in clinical symptoms (as captured by clinical assessments or self-report scales: MADRS, GAD-7, and PHQ-9); remission is defined as clinical assessment scores below the clinical threshold (e.g., MADRS $\leq$ 10); and relapse is characterized by the recurrence of symptoms after a patient has achieved remission (e.g., MADRS $\geq$ 20 after initial remission).[32]

There are concerns that the use of digital platforms may affect the therapeutic relationship, yet there is a paucity of studies exploring this impact. A prior study focused on the effects of Information & Communication Technologies (ICT) in two culturally distinct countries, and the authors reported that the increased deployment of ICT facilitated patient empowerment.[33]

Similarly, in a systematic review of 37 studies of patients with various health conditions, all of them reported a beneficial impact of digital platforms on the therapeutic alliance, including: improved communication, interaction and clinical discussion; clinicians' feelings of compassion and empathy; and patients' feelings of reassurance, comfort, connection, trust and accountability. Only two studies reported negative effects, such as patients' feelings of isolation and therapists' concerns of violation of therapeutic boundaries.[34]

As for the impact of digital platforms on mental health, another systematic review of five studies found that one study reported that the patient-therapist alliance was enhanced, two did not find any difference, and two reported that app engagement and acceptability depended on the therapeutic relationship. Most importantly, none of them reported harmful effects. Due to the substantial heterogeneity in the methodology of the studies included in this review, the interpretation of its results are limited. However, in general, the studies included in this review agreed that digital platforms, such as smartphone apps, can act as adjuncts and support an increase in engagement and treatment adherence.[35] Two other systematic reviews on mental health apps reported that dropout rates tended to be lower in studies offering human feedback and interventions such as in-app self-monitoring.[36,37]

2.2. Objectives

This project aims to determine the feasibility of utilizing digital platforms to collect active and passive data related to mental health symptoms among adult outpatients with TRD receiving clinical treatment at the IPP and evaluate the association between the digital data collected with the treatment outcomes of relapse, remission and response. The proposed digital platforms include (1) a wearable device (Oura Ring)[39] to capture passive data on activity, sleep and physiological parameters, and (2) a web-based clinical data management platform (REDCap[38]) to capture responses to validated clinical scales (PHQ-9 and GAD-7) and clinical assessments (MADRS, intake form, demographics) conducted as part of patient care.

The REDCap platform will be available to all participants enrolled in this study; this platform will enable the clinical team to record participants' self-reported symptoms in an electronic platform outside the medical electronic health records system for use in research. Oura Ring use

will be optional and will depend on: 1) device availability, 2) participant's interest, and 3) participant's ownership of a smartphone compatible with the Oura App (to sync and visualize data). We anticipate that approximately 60 Oura Rings will be available for participants to use. Participants will be able to visualize their passively captured data (e.g., activity, sleep parameters, heart rate, respiratory rate, body temperature) on the Oura App in near real-time. These data streams have the power to enable a fine-grained understanding of a patient's experience through means of continuous data collection, visualization, and self-monitoring.

This study has the following aims:

- **Primary Aim:** To examine the feasibility of a digital suite (REDCap clinical scales and a wearable device; Oura Ring) to collect active and passive data, respectively, among adults receiving clinical treatment at the IPP for TRD.
- **Secondary aims:**
    - To evaluate the association of active data (from REDCap) and passive data (from the Oura Ring) over the duration of clinical treatment with the outcomes of treatment response (reduction of ≥ 50%), remission (score ≤ clinical threshold), and relapse (score ≥ clinical threshold after initial remission).
    - To create personalized digital phenotype profiles (pDPPs) based on wearable and web-based data.

2.2.1 Personal Digital Phenotype Profile Definition

We propose the definition of digital phenotype profile (DPP), which calculates an individual's baseline and continuously monitors for changes in DPP characteristics derived from mathematical models. Digital phenotype profile (DPP) is a representation of a user's physical and behavioural health using the baseline data collected at the beginning of the study as reference. The models are then fine-tuned with the continuous incoming data streams. However, variations of the model representations require further investigation to ensure its accuracy and robustness. This work investigates the statistical aspect of the analysis pipeline to offer a robust DPP representation. Specifically, the use of robust principal component analysis (RPCA) to extract sparse representation. The DPP is developed through our Statistical, Information Theory,

and Data-driven (SID) pipeline, then is represented through the sparse-rank matrix using RPCA. Once the DPP representation has been created, we additionally enhance this definition by proposing the term pDPP as a personalized version of the DPP, where we use collected longitudinal data to monitor the changes in physical and behavioural health of an individual participant.

The process of digital phenotyping involves building a mathematical model using the active and passive data collected from each participant. An individual model is built for each person by incorporating their physiological data together with their self-reported affective states collected from questionnaires. Once the personal profile/model has been constructed, physiological data (such as that collected from the Oura Ring) can be used to provide insight into a person's affective state, even without any active data. In the long run, such models could allow for insights into patients' mental well-being in a non-intrusive way that can improve mental health care and responses to stress.

3. METHODS

3.1. Study Design

This observational study with retrospective data analysis will assess the feasibility of multimodal digital platforms to collect active (self-report) and passive (wearable biometrics) data and enable self-monitoring of adult patients receiving clinical treatment for TRD. Specifically, we will assess the feasibility of using a web-based electronic data capture platform (REDCap[38]) and a wearable device (Oura Ring[39]) to collect active and passive data, respectively, in tandem with neuropsychiatric treatment for TRD in the IPP. The overall duration of this study is three years. However, each participant will only be enrolled in this study for the duration of their clinical treatment in the IPP. All participants will begin participation during the first study visit (known as the Screening and Baseline Visit), which will take place upon scheduling of the first treatment session. For participants receiving the Oura Ring, the Screening and Baseline Visit will occur in-person (so they can obtain an Oura Ring) and will take place approximately three weeks before the first treatment session, to establish baseline parameters measured by the Oura Ring. For participants not receiving the Oura Ring, their clinical treatment can begin after the

Screening and Baseline Visit (usually ~0-3 weeks after), therefore, some participants may complete these measures at their first treatment session .

Following REB approval, 200 patients aged 18 years or older will be recruited over 33 months and will have their self-report assessments (GAD-7 and, if applicable, PHQ-9) entered on the REDCap platform for use in research (see Appendix A). Depending on the availability of Oura Ring devices, a subset of participants will also be offered the opportunity to use an Oura Ring, which is a wearable device that will collect data on sleep, activity, and physiological data in near real-time during their participation in this study. We estimate that approximately 60 Oura Ring devices will be available for participants to use. Potential participants will be adults with TRD enrolled for clinical treatment at the IPP at St. Michael's Hospital, Unity Health Toronto (SMH-UHT). Participants will receive one of the clinical treatments at the IPP according to clinical protocols and standard of care (Figure 1). The details of the study design are summarized in Figure 2.

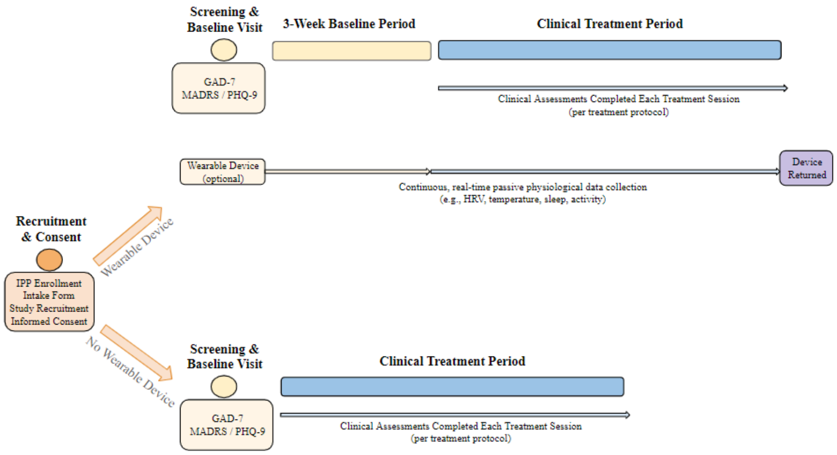

Figure 2: Study design. Upon enrollment to the IPP, all participants will complete an intake form (including demographics and medical history), will be allocated to one IPP treatment arm based on the intervention decision hierarchy, and informed consent to participate in digital data collection will be obtained. For participants eligible and interested to receive an Oura Ring, they will complete an in-person Screening and Baseline Visit approximately three weeks before the first treatment session to receive their wearable device and establish a reliable physiological baseline prior to the onset of treatment. Participants not receiving an Oura Ring can begin study participation on the first day of treatment. Duration of treatment will be dependent on the type of intervention participants are receiving, in accordance with standard administration protocols.

3.2. Eligibility Criteria

3.2.1. Inclusion Criteria

This study will adopt the following inclusion criteria (Appendix C):

1. Adults ≥18 years of age who are capable of giving informed consent
2. Patients meeting diagnostic criteria for major depressive disorder (MDD) without psychotic symptoms according to the Diagnostic and Statistical Manual of Mental Disorders – Fifth Edition (DSM-5)[1] and currently experiencing a Major Depressive Episode (MDE) as confirmed at intake
3. A Montgomery-Asberg Depression Rating Scale (MADRS)[3] score ≥ 20 at screening (moderate-to-severe MDE)
4. Failure of two or more adequate trials of antidepressant therapy of adequate dose and duration during the current episode as confirmed at intake
5. Undergoing clinical treatment at the Interventional Psychiatry Program at St. Michael's Hospital, Unity Health Toronto (SMH-UHT)
6. Ownership of a smartphone with Bluetooth capabilities (for participants using the Oura Ring)

3.2.2. Exclusion Criteria

This study will adopt the following exclusion criteria (Appendix C):

1. Subjects without internet access, as an internet connection is required for using the study platforms
2. Medication changes, aside from the treatment received through the IPP, one month (28 days) before screening, or during the entire duration of treatment (variable based on treatment arm).
3. Other exclusion criteria for this study will follow those from the clinical treatment the participant is receiving (Standard of care)

Participants without a smartphone with Bluetooth capabilities will not be eligible to receive the Oura Ring, as they must be able to download the Oura App (for data syncing and visualization).

However, these participants can still contribute data to the REDCap components of the study. Non-English-speaking individuals are excluded because it is necessary for participants to have the ability to communicate and understand study information, answer questions accurately and completely about the study, obtain informed consent, and complete self-report measures independently.

3.3. Withdrawal Criteria

Participants are free to withdraw their consent and/or end their participation in the research study at any time without penalty or loss of benefits or treatment to which they are otherwise entitled. Participants may be withdrawn from the study by the study's doctor if they feel that it is in their best interest.

Participants may also be withdrawn if:

- The participant meets any exclusion criteria for this study
- The participant meets any exclusion criteria for the clinical treatment they are receiving within the IPP (either newly developed or not previously recognized)
- The participant stops receiving clinical treatment within the IPP
- Participant fails to adhere to study responsibilities
- They must be withdrawn from clinical treatment for any reason
- Anything, in the opinion of the investigator, that would place the participant at increased risk or preclude the participant's full compliance with or completion of the study.

If a participant withdraws from the study at any time, the reasons for withdrawal will be collected and documented as part of the feasibility outcome for this study. A time will be scheduled for the participant to return the Oura Ring (if applicable). Data collected up until the point of withdrawal will be used for analyses.

3.4. Concomitant Medications

Participation in the study will not alter the standard of care, including any required changes to treatment regimens. Medications will be monitored on a regular basis and may be restricted as part of the standard of care for their respective clinical treatment. Participants will be required to

continue with their respective approved treatment for MDD throughout the study. Before the intervention, investigators will analyze all medications taken by participants on a regular basis. No medication changes, aside from the treatment received through the IPP, are allowed one month (28 days) before screening, or during the entire duration of treatment (variable based on treatment arm) and follow-up (14 days) due to the probable impact on brain activity and psychiatric symptoms. Participation in the study will not alter the standard of care, including any required changes to treatment regimens. If any changes to concomitant psychotropic medications are required as per the patient's standard of care, the participant will be withdrawn from the study.

3.5. Concomitant Care

Regular psychiatric care will be provided by the participant's primary responsible physician without restriction.

3.6. Interventions

Details on the active (self-report clinical scales) and passive (wearable device) digital interventions are described below.

All data collection will be de-identified, such that participants will be assigned a unique identification (ID) number and de-identified dummy email address. The study ID will be entered into the REDCap platform and used for all CRFs, in lieu of personally identifying information. The de-identified email address and associated password will be used to set-up participants' wearable app accounts and will be used for email forwarding (to participants' personal email addresses) to ensure participants remain de-identified on the Oura platform.

The Study Coordinator will manage recruitment, informed consent, and liaise with the clinical team throughout the course of treatment. The clinical team will enter patient-participant data directly into REDCap (based on intake and CRFs collected as part of clinical care in the IPP) so as to simply observe the clinical process (not intervene) and minimize data loss. A member of the research team (engagement specialist) will monitor wearable data compliance on a weekly basis; if a participant's Oura data is missing for 3 or more days (over the past 7 days), the

clinical team will be notified so they can connect with participants and refer them to the research team to troubleshoot any technical issues as needed. The clinical team will provide regular reminders at in-person treatment sessions to charge their wearable device and regularly synchronize, and visualize, their passive data in the smartphone app.

3.6.1. REDCap

Participants will complete mental health related self-report questionnaires as part of their clinical care; the specific scales and frequency to be completed will be dependent on IPP clinic process and will vary across treatment arms (e.g., rTMS patients complete the GAD-7 and PHQ-9 for each treatment session, IVK patients complete the GAD-7 and MADRS at each treatment session). The clinical team will enter patient-participants' questionnaire responses directly into the REDCap[38] platform after the treatment session (Appendix A). 3.6.2. Oura Ring Wearable Device

We anticipate approximately 60 Oura Rings will be available for participants in this study. Participants who have a smartphone compatible with the Oura App and understand the requirement of returning the wearable device at the end of the study will be eligible for receiving the Oura Ring. Depending on availability, participants will be offered a commercially available wearable device to use for the duration of the study to collect passive data that is relevant to mental health and well-being such as physiological data, sleep and activity patterns. The Oura Ring is a wearable device that can be used without obstructing one's daily life. Detailed information on the features to be collected by the Oura Ring are summarized in Section 3.8.4.2.2 (Table 1). The Oura Ring device has its own mobile app for data visualization and self-management.

The Oura Ring has been successfully used in previous research studies to provide information on the behaviour of body systems (e.g., autonomic nervous system, sleep/circadian rhythm) in real-time through accurate measures of physiological parameters, sleep and activity.[25,40,41] A recent study using the Oura Ring reported significant associations between depressive symptoms and sleep features, with the combined use of the ring, mobile app, and self-reported mood providing the strongest prediction of depression.[25] In the proposed study, we will use Oura Ring

to continuously collect activity, sleep, and physiological signals for retrospective modeling and integration with active (clinical assessment) data to more precisely monitor a participant's activity, behaviour, and wellbeing as compared to either of these platforms alone. The description of the physiological data to be collected by the Oura Ring is provided below as follows:

1. Sleep information: Oura Ring performs sleep analysis and stores a set of measurement parameters that summarize each period. The ring calculates the sleep period specific parameters within four hours from the period end, but sleep analysis is always triggered when you open the application. These parameters include bedtime, total duration, awake, light, Rapid Eye Movement (REM), and deep sleep durations, and also heart rate, breathing, temperature changes during sleep.
2. Activity information: Activity summary contains daily activity summary values and detailed activity levels. Activity levels are expressed in metabolic equivalent of task minutes (MET mins). Oura tracks activity based on the movement and presents the activity score based on duration of low, medium, and high activity levels. The movement is also used for step count and to estimate calories.
3. Readiness information: Readiness score is interpreted from sleep, activity, resting heart rate, heart rate variability, recovery index and temperature scores. A Readiness Score above 85% indicates that you're well recovered. A score below 70% usually means that an essential Readiness Contributor, such as body temperature or previous night's sleep, falls outside your normal range, or clearly differs from recommended, science-based values.

The wearable device privacy policy will be made available for all potential users to read. Data collection via the Oura Ring for selected participants will start approximately three weeks before the clinical treatment begins, continuing throughout the intervention and ending on the last day of treatment. Oura Rings will be returned to the clinical team at the end of their last treatment session.

Data collected via Oura will be de-identified using a de-identified study ID and email address which will be used to login to their account on the Oura app. This will ensure that participants are de-identified on this third-party platform (Oura).

A member of the research team will monitor data compliance on a weekly basis. If participants have Oura data that are missing for 3 or more days over the past 7 days, the clinical team will be notified so they can follow up with participants. Participants will be referred to the research team as needed to troubleshoot any technical issues.

3.7. Outcomes

All outcomes will be assessed at different stages of the study. Please refer to the study's schedule, section 3.8.1, for a detailed depiction of the nature and frequency of all assessments.

Primary Outcome:	Feasibility of REDCap and the Oura Ring will be measured by:<br>1) Number of recruitments (minimum threshold: 6-7 participants/month)<br>2) Recruitment rates (proportion of recruited participants over the total number of patients invited for this study)<br>3) Dropout rate (maximum threshold: 30%) and reasons for dropping out<br>4) Data completion rates (minimum threshold: 70%)<br>Data will be compared among responders versus non-responders as well those who do and do not relapse and/or remit
Secondary Outcome	Clinical outcomes will be measured by:<br>a) Quantifying changes in active (self-report) and passive (physiological) data over time<br>b) Predicting treatment response , remission, and relapse based on active (self-report) and passive (physiological) data .

	c) Constructing personalized digital phenotype profiles (pDPPs) based on wearable and web-based data.

3.8. Procedures

3.8.1. Study Schedule

	**First Study Visit (Screening and Baseline Visit)**	**Baseline Period (approx. 3 weeks)***	**Intervention (Length depends on treatment)**
**Frequency of each Measure**		**Daily**	**Treatment Session**
Oura Ring (~60 rings available)	✔	✔	✔
Intake Form	✔		
**REDCap Data Collection**			
GAD-7	✔		✔
PHQ-9	✔		(✔)
MADRS	✔		(✔)

GAD-7 – Generalized Anxiety Disorder – 7-item Scale

PHQ-9 – Patient Health Questionnaire – 9-item Scale

MADRS - Montgomery-Asberg Depression Rating Scale

✔ - Assessment or data collection relevant to all treatment arms.

(✔) - Assessment included in some treatment arms but not all.

Note: As per IPP clinic protocol, patients undergoing rTMS will have their GAD-7 and PHQ-9 self-report responses entered after each treatment session, whereas patients undergoing IVK or ECT treatment will not complete the PHQ-9 (i.e., only the GAD-7 will be entered on REDCap) and will instead complete the MADRS with a member of the clinical team at each treatment session.

3.8.2. Recruitment

Patients who have been referred to the IPP to receive rTMS, IVK or ECT treatment will be recruited to participate in this study. An individual from the program's clinical team will inform newly referred patient-participants that either one or two digital platforms – the REDCap platform and (if eligible and available) the Oura Ring wearable device – are available to facilitate the collection of data. The collection of active data will allow researchers to evaluate longitudinal changes in self-report cognitive-behavioural symptoms; passive data will allow participants to monitor their own data in real-time which can have a positive impact on their health since it can promote healthy behaviours. If patients are interested in participating, the clinical team will direct potential participants to a member of the research team, who will provide more information about the study and obtain informed consent (see Appendix E).

3.8.3 Informed Consent

Potential patient participants who are interested in the study will be emailed an informed consent form (ICF) and contacted by a member of the research team to discuss study details and answer any questions the participant may have. The ICF will clearly state to interested patients that their data will be de-identified for the wearable device (Oura Ring) and that the study team is not able to discuss any mental health concerns with them. Participants will have the opportunity to discuss any questions about the study with the Study Coordinator. They will also have the option to speak with the Principal Investigator, whose contact information will be provided in the ICF. Once the patients have carefully read through the ICF, they will then be asked to confirm their interest and return a digitally signed copy to the research team. A member of the research team will then verify and confirm each patient's enrollment and eligibility.

3.8.4 Screening and Baseline Visit (30 - 60 Minutes)

Upon receiving digital consent, the first visit (which will constitute a Screening and Baseline Visit) will be scheduled by the clinical team. Participants will sign a hard-copy of the ICF in-person, complete baseline measures of general anxiety (Generalized Anxiety Disorder Scale, GAD-7[42]) and depression (Patient Health Questionnaire, PHQ-9[43]; or Montgomery-Åsberg Depression Rating Scale, MADRS[3]), and their Oura account will be set-up(if applicable). For participants receiving an Oura Ring, the Screening and Baseline Visit will take place in-person (at the Interventional Psychiatry Program at St. Michael's Hospital) at least three weeks before the start of their clinical treatment so they can receive a properly-fitting device and complete a baseline recording period to establish a reliable baseline for passive data collection and Oura's app features. More specifically, a member of the study team will determine the participant's ring size (using an Oura sizing kit); provide the appropriate device; assist participants in downloading, setting up, and using the Oura app on their smartphone using their assigned de-identified credentials (de-identified email address and password); and answer any questions the participant might have. In contrast, this visit will occur on the first treatment day for participants who are not receiving the Oura Ring (i.e., REDCap only), as part of clinical care.

3.8.5 Intervention

3.8.5.1 Standard of Care

Participants will receive the standard treatment protocol for one of the clinical treatments offered at the IPP for TRD (rTMS, IVK, and ECT). During clinical visits, participants will complete self-report questionnaires (GAD-7, PHQ-9), and may complete a clinician-administered assessment of depression (MADRS), as part of clinical care; assessment administration and frequency in the IPP is dependent on the treatment arm. The clinical team will enter participants' responses to these measures into REDCap after each treatment session. The Columbia-Suicide Severity Rating Scale (C-SSRS) may also be collected by the clinical team to continuously assess for suicidality while participants are in treatment; however, the C-SSRS will not be used as an outcome measure for this study.

3.8.5.2. Digital Intervention Data Collection

3.8.5.2.1. Active Data – Self-Report Questionnaires via REDCap

For active data collection, a member of the clinical team will enter patient-participants' self-report questionnaire and (if applicable) clinical assessment responses directly into the REDCap platform at the end of each study visit and treatment session (Appendix A). Active data will be used in combination with passive data – activity, sleep, and physiological data – for correlation and prediction of the clinical landmarks of response, remission, and depressive relapse.

<u>As per IPP clinic process, participants will complete the following assessments on each treatment session day (approximately 10 - 30 minutes):</u>

1. Anxiety: Generalized Anxiety Disorder - 7-item scale (GAD-7)
2. Depression: Patient Health Questionnaire- 9-item scale (PHQ-9) or Montgomery-Asberg Depression Rating Scale (MADRS)

3.8.5.2.2. Passive data through Oura Ring

During the Screening and Baseline Visit, a member of the study team will familiarize participants with the Oura Ring and mobile application. Participants will use the credentials of a de-identified email account to log in to the Oura Ring application. Participants will be asked to wear the Oura Ring throughout their involvement in the study (3-week baseline + IPP treatment). Table 1 provides details of the parameters collected by the Oura Ring.

Table 1: Description of the Oura Ring wearable device and data collection

Device	Description	Data Transfer	Data elements
Oura Ring	The Oura Ring is a smart biosensor device that is worn on the finger and passively collects physiological parameters and information on sleep and activity levels of an individual. It is considered a smart-ring because it uses advanced sensor technology to allow for precise, personalized health insights about one's body (see Data Elements).	Participants will download the Oura app to their smartphone and log on using their de-identified study ID details. The Oura Ring must be connected to the smartphone via Bluetooth to regularly sync data. The Oura Ring collects data locally and sends it to the mobile app when synchronized. Once the Oura App is connected to the internet (e.g., wifi or phone data), the data will be uploaded to the remote Oura server. For data analysis, only authorized research members will have access to the data collected by Oura Ring through its respective application programming interface (API).	Some of the physiological and activity data extracted from the Oura Ring include:<br>● Heart rate<br>● Heart rate variability (HRV)<br>● Respiration rate<br>● Daily activity levels<br>● Oxygen saturation (SpO2)<br>● Body temperature variability (delta)<br>● Readiness score<br>● Calories<br>Sleep data:<br>● Sleep stages: deep sleep, rapid eye movement (REM), light sleep and awake periods<br>● Sleep score<br>● Nap detection

The following steps will outline the data collection process of the Oura Ring device:

1. The account and device will be set up by a study team member with a unique de-identified study ID and email. The data will be anonymous and only identifiable by select research members with access to the master log.
2. Metrics extracted by the Oura Ring will be collected and synchronized to the Oura smartphone app (via Bluetooth) and the Oura server (via secured internet connection). Oura has its own secure server where the data will be stored separately from active (REDCap) data.
3. The research team will have access to the secured Oura Application Programming Interface (API) to extract data from its respective server. The API will securely connect to the respective servers to extract de-identified passive data. The de-identified data will be available to the research team for analysis. The data will be securely extracted using the Oura API and stored on an external encrypted and password-protected hard-drive, accessible to only select members of the research team. The data extraction program will be executed weekly so that data will be extracted and stored regularly.

Since participants who use the Oura Ring for this study will be undergoing different treatments (rTMS, IVK or ECT), there is a possibility that wear times may vary slightly according to the duration of clinical treatment, and this will be the same for REDCap data collection. The device will be returned to the study team at the end of the last treatment session. All returned rings will be cleaned, sanitized, and allocated a new de-identified study ID before being given to another participant.

3.9. Reimbursement

There are no reimbursement costs associated with this study, as all study activities are taking place alongside IPP clinic treatment.

3.10. Quality Assurance

Methods for quality assurance will be strictly observed. A start-up meeting involving all study site research staff, co-investigators, and partners will be held prior to the launch of the study.

Further training (e.g., REDCap, Oura sizing and use) will be held prior to commencing the study. During training, great care will be taken to fully explain the clinic and study procedures and case report form (CRF) completion.

4. DATA MANAGEMENT

Active data collected through the REDCap platform will be stored on the REDCap server (accessible to the study team). Demographic and medical history data collected as part of the IPP intake process will be stored on the UHT Network folder and patients' electronic health records (EHRs; accessible only to the clinical team). Paper questionnaire forms used as part of clinical care will be stored in a locked cabinet accessible only to the clinical team and will be securely destroyed after being entered into the patient-participant's REDCap form and EHR. The Oura Ring will collect de-identified passive and continuous data through wearable technology. The data will be collected on the respective app, whereform it will be transferred to and stored on its respective server. The digital platform will be set up to comply with the protection of privacy of the participant. We will use the Oura API to extract available data from their servers and store on an external encrypted and password-protected hard-drive. Data collected from the Oura Ring and REDCap will be independent from one another and will only be linked for analysis. No personal health or clinical information will be shared with Oura.

A designated research team member will ensure that the REDCap and Oura platforms are working properly by surveilling the platforms and data on a weekly basis. The clinical team will be notified of any participants with missing data over the past week (3 of the past 7 days) so they can communicate with the patients at the next treatment session. Participants will be referred to the research team to troubleshoot any technical issues, as needed. Participants will be able to contact the Study Coordinator (Karisa Parkington, karisa.parkington@unityhealth.to) with any study-specific questions throughout the study duration, or may contact the study-specific email (IPPRegistry@unityhealth.to) - which is monitored daily by the research team - for technical assistance.

4.1. Security and Privacy of the Study Data

4.1.1. Oura Ring Wearable Device

A subset of participants will use the Oura Ring wearable device to collect passive data (Section 3.6.2). The device and its respective app will have a specific user ID and de-identified email that will be set up by the research team so that the participant remains de-identified on this platform. Data collected from the Oura Ring wearable device will be made accessible through the Oura API. Each participant's de-identified data will be exported and stored on an external encrypted and password-protected hard-drive and the data will only be associated with a participant identification number.

Each device will collect parameters related to mental health and wellness, such as heart rate variability (HRV), sleep metrics, and the Oura Activity Score. Additionally, the Oura device and mobileapp are independent from the web-based REDCap platform; therefore, data from one platform are not linked or related to any other platform except through the de-identified ID assigned to the participant.

No personal information will be collected through the Oura digital platform and only authorized members of the project will be able to access the data. Study data will be stored for a minimum of seven years and may be used for other research or analyses by the investigators, or by other researchers. Future studies where this data will be used or shared will be subject to REB approval.

4.1.3. REDCap

REDCap (https://www.project-redcap.org) is supported by the Applied Health Research Centre (AHRC) at UHT. REDCap is an open-source, web-based clinical data management and electronic data capture system and database. The system is developed and managed in compliance with UHT privacy, the Health Insurance Portability and Accountability Act, the Personal Information Protection and Electronic Documents Act (PIPEDA), and Food and Drug Administration 21 Code of Federal Regulations Part 11 regulations, providing functions such as defined user roles and privileges, user authentication and encryption for in-transit data, de-identification of protected health information, and comprehensive auditing features to record and monitor access and changes to data. This system will be used to collect and store active

(CRFs and self-report questionnaire) data and for the query and export of datasets for statistical analysis and modeling.
Access to REDCap will be protected through a secure web portal and protected by multiple levels of authentication and authorization. Project Coordinators will be assigned the project administrative privileges for study configuration, data collection management, and quality control. Participants will complete self-report mental health questionnaires in line with their clinical treatment protocol; upon acceptance to the study, the clinical team will enter patient-participants' responses into REDCap after each treatment session (based on CRFs completed as part of clinical care). Active data will be stored on the REDCap server and will be linked by the de-identified ID for final analysis. Only Project Administrators will be granted access to download the data from the REDCap server for analysis.

During data analysis, de-identified data will be provided to the analysts. This will be achieved using a two-zone approach. The two-zone approach divides study team members into two groups; identified and de-identified. As the names suggest, the identified group will have access to identified (i.e. demographic data) and de-identified information and thus is able to re-identify the data to provide data management, quality and data linkage functions (on active and passive data) whereas the de-identified group will only have access to the de-identified information. This will ensure that the data analysts of the team only have access to de-identified data but will not have access to any of the participant's identifiers to protect participant privacy.

5. DATA ANALYSIS METHODS

5.1 Statistical Analysis

We will assess the feasibility of digital interventions using descriptive statistics with counts and proportions for categorical data and means and standard deviations (parametric) or median and interquartile range (non-parametric), as appropriate, for continuous data. The proportion lost to follow-up will be estimated along with a 95% confidence interval. The upper 95% confidence limit should not exceed 20% to proceed. The proportion compliant with the protocol, including treatment compliance as well as study completion and complete data on clinical outcomes (i.e., the per-protocol group) will be estimated with a 95% confidence interval. For validity, this

should be fairly high so that the lower 95% confidence limit is > 80%.Group- (*t*-tests, ANOVA, ANCOVA, correlation, regression) and individual- (multilevel modeling) level biostatistics will be used to identify and quantify changes in active (anxiety and depression symptoms) and passive (biometrics) data across treatment arms and over time.

Machine learning and advanced analytics (e.g., dimensionality reduction, statistical relevance or multi-view bi-clustering) will also be used to fuse the passive physiological data with the active questionnaire data. More specifically, passive data is effective at identifying behaviours and trends in activity but is poor in measuring people's internal states, motivation, and attitude, whereas active data is the opposite. Developing a methodology for integrating the two data sources can mitigate their respective weaknesses. The passive physiological data will be fused with the active data collected by the REDCap platform. The motivation to fuse active and passive data includes the cross-validation and improvement of measurements, the explanation of human behaviour, and novel opportunities to improve causal inference in experimental settings.

The active data will serve as a ground truth to validate the passive data from the mobile phone for monitoring a "depressed experience". We will use generalized machine learning models to detect and predict distress and depression among participants and develop personalized temporal machine learning models for personalized application. This will involve creating predictive models based on a participant's data and implementing the model suited for the participant. This proposal will be an iterative process. To combat missing data, filtering, and encoders can be used to interpolate and predict the expected data accordingly.

During the analysis, we will extract features from the passive physiological data for better representation. Trends, classifications and relationships will be determined using different machine learning techniques. Implementations of pre-processing, feature extraction and machine learning will be conducted in a numerical program for efficient and powerful processing that will achieve impactful results.

5.2. Personal Digital Phenotype Analysis and Generation

Our goal is to have a systematic investigation of DPP with an emphasis on robustness for long-term utility. We envision the DPP to be represented as a multi-dimensional vector composed of passive and active data, where each profile is unique to one another. To do so, we propose a Statistics, Information Theory, and Data-driven (SID) pipeline to develop the foundation of the DPP. The proposed SID pipeline is encapsulated in Figure 3.

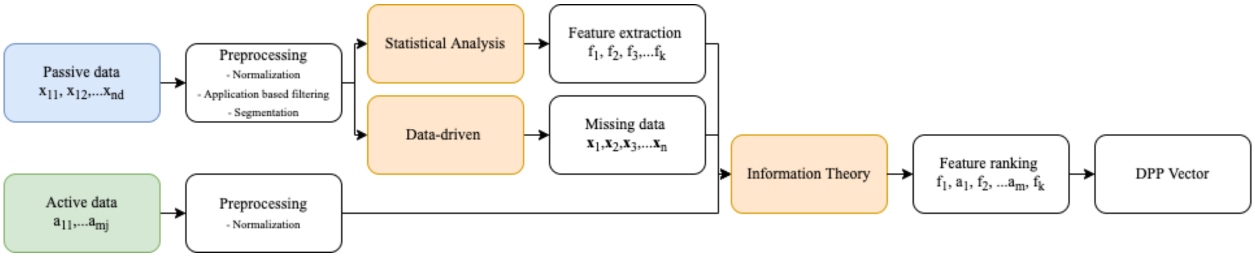

Figure 3: Block diagram representation of the proposed SID pipeline

DPP will be created through the use of SID, and will be a representation for each individual user.[50] Once the DPP is created, we enhance the analysis by proposing the personalized digital phenotype profile (pDPP) where we develop individual models for each person by incorporating their physiological data together with their self-reported affective states collected from questionnaires. Once the personal profile/model has been constructed, physiological data (such as that collected from the Oura Ring) can be used to provide insight into a person's affective state, even without any active data. The pDPP will be used for long-term utility where we monitor the changes in physical and behavioural health of an individual participant. The application of pDPP could allow for insights into mental well-being in a non-intrusive way that can improve mental health care and responses to stress.

5.3. Interpretation of the Personal Digital Phenotype Profile

Our goal is to have a systematic investigation of pDPP with an emphasis on robustness for long-term utility. We envision the pDPP to be represented as a multi-dimensional vector composed of passive and active data, where each profile is unique to one another. We will use analytical techniques such as robust principal component analysis (RPCA) to represent the data. In addition, RPCA decomposes the matrices to low- and sparse-rank representation where the

low-rank representation can be considered the outliers and sparse-rank can be considered as the concise representation of the pDPP vector. We plan to develop an algorithm to analyze the low-rank representation for anomaly detection where new additional samples are added (passive or active data) or when the pDPP baseline varies past an accepted threshold, an alert of potential change in physical and behavioural health can be announced.

5.4. Sample Size Considerations

This project aims to collect pilot data on the feasibility of the REDCap platform and the Oura Ring wearable device to collect data of adult outpatients receiving clinical treatment for TRD; therefore, a sample size of 200 participants should provide enough power for pilot quantitative analysis across treatment arms. We have followed the sample size calculator for digital phenotyping studies developed by Barnett et al.[51]

6. ETHICS AND DISSEMINATION

6.1. Research Ethics Considerations

This study will be conducted in accordance with the ethical principles laid down in the Declaration of Helsinki, the protocol, Good Clinical Practice guidelines, and applicable privacy legislation, include the Personal Health Information Protection Act (PHIPA) of Ontario, and The Personal Information Protection and Electronic Documents Act (PIPEDA). We will obtain approval from the Research Ethics Board at St. Michael's Hospital before any study activities commence. Full written informed consent will be obtained prior to conducting any study activities.

6.2. Protocol Amendments

Before any changes to the study are implemented, besides those to eliminate immediate hazards to study participants, an amendment to the study will be reviewed and approved by the REB.

6.3. Informed Consent Process

Patients will be recruited from the IPP at SMH-UHT.Patients who meet the inclusion criteria and have first consented to receive a standard clinical treatment – rTMS, IVK or ECT – as part of

their care (i.e., signing the treating hospital's corresponding consent form) will be informed about the study and directed to a member of the research team, if interested. A member of the research team will provide them with information about the purpose of the study. The participants will be emailed the ICF, given sufficient time to read the ICF and ask any questions before deciding to participate. Participants who consent to participate in the study will then be asked to sign the electronic ICF. These recruitment and consenting procedures will be carried out before the psychiatrists and anesthetists provide clinical care to the study participants.

6.4. Exclusion of Women, Minorities, and Children (Special Populations)

Non-English-speaking individuals are excluded because the ability to accurately and completely communicate study information, answer questions about the study, and obtain consent is necessary. Those without internet connection will not be eligible to receive a wearable device (because internet access is required for data synchronization with the wearable servers) but can contribute to the REDCap data collection. Participants who opt to use the Oura Ring must also own a smartphone in order to transfer the data collected by the Oura Ring to the respective app and servers.

6.5. Confidentiality

Participant confidentiality is strictly held in trust by the participating investigators, their staff, and the research team. This confidentiality is extended to cover the clinical information relating to participants. All data will only be referenced by a de-identified numerical identifier code and will be stored on a password-protected computer. A key connecting names and code numbers (i.e., Master Linking Log) will be kept in a password-protected document on Citrix, accessible only by authorized research personnel. Participants will not be personally identified in any report or publication about this study.

6.6. Study Discontinuation

In the event that the study is discontinued, subjects who have completed or who are still enrolled in the study will be notified. Any new information gained during the course of the study that might affect subjects' safety or willingness to continue participation in the study will be

communicated to participants by a member of the research team within two days after the Principal Investigator learns this information.

6.7. Declaration of Interests

The principal investigators of this trial have no financial or competing interests to declare.

6.8. Dissemination Policy

The results of this trial will be published in a peer-reviewed journal and presented at scientific conferences/meetings.

6.9. Authorship

To be eligible for authorship on any resultant publications, all potential contributors must fulfill all criteria as set forth by the International Committee of Medical Journal Editors.

7. IMMEDIACY & IMPORTANCE OF RESEARCH IMPACT

This study will be the first of its kind to investigate the feasibility of implementing multimodal digital interventions alongside neuropsychiatric treatment (rTMS, IVK, or ECT) for TRD. This knowledge will generate unique insights into accessible digital interventions for adults with TRD, which will support current evidence and prompt further examination of efficacy of digital interventions in larger studies. Pilot data on clinical outcome prediction (remission/response/relapse) will provide additional insights into more definitive studies to determine the efficacy of digital platforms for monitoring response to depression treatment. In the long term, the determination of the effectiveness of digital interventions in TRD would aid in the expansion of their use to other mental illnesses and enable real-time measurement-based care, which could enhance clinical decision-making, improve patient outcomes, and prevent illness relapse.

8. STUDY ADMINISTRATION

8.1. Key Contacts

Study Principal Investigator

Venkat Bhat, MD MSc FRCPC DABPN
St, Michael's Hospital and the University of Toronto
Venkat.bhat@unityhealth.to
416360400 Ext 76404

Study Coordinator

Karisa Parkington, PhD
St. Michael's Hospital
karisa.parkington@unityhealth.to

Study Co-Investigators

Perry Menzies, MD MSc FRCPC
St. Michael's Hospital and the University of Toronto
Perry.menzies@unityhealth.to

Sridhar Krishnan, PhD Peng FCAE
Signal Analysis Research Lab and Toronto Metropolitan University
(416) 979-5000 x554931
krishnan@ryerson.ca

Wendy Lou, PhD
Dalla Lana School of Public Health, University of Toronto
416-946-7804
Wendy.lou@utoronto.ca

Study Staff

Fathima Adamsahib, BSc, PA-C
St. Michael's Hospital

Reinhard Janssen Aguilar, MA, MD
St. Michael's Hospital

Gyu Hee (Sarah) Lee, BSc
St. Michael's Hospital

8.2. Funding

Miner's Lamp Innovation Fund in Prevention and Early Detection of Severe Mental Illness; Department of Psychiatry, University of Toronto.

8.3. Roles and Responsibilities

8.3.1. Sponsor and Funding

The study funder has no role in study design, collection, management, analysis, and interpretation of data; writing of the report; and the decision to submit the report for publication.

9. REFERENCES

1. American Psychiatric Association. *Diagnostic and Statistical Manual of Mental Disorders*. 5th ed. American Psychiatric Publishing, Inc.; 2013.
2. Sheehan DV, Lecrubier Y, Sheehan KH, et al. The Mini-International Neuropsychiatric Interview (M.I.N.I): The development and validation of a structured diagnostic psychiatric interview for DSM-IV and ICD-10. *J Clin Psychiatry*. 1998;59:22-33.
3. Montgomery SA, Åsberg M. A New Depression Scale Designed to be Sensitive to Change. *Br J Psychiatry*. 1979;134(4):382-389. doi:10.1192/bjp.134.4.382
4. Dew RE. Adequacy of Antidepressant Treatment by Psychiatric Residents: The Antidepressant Treatment History Form as a Possible Assessment Tool. *Acad Psychiatry*. 2005;29(3):283-288. doi:10.1176/appi.ap.29.3.283
5. World Health Organization. *Depression and Other Common Mental Disorders: Global Health Estimates*. World Health Organization; 2017. Accessed February 8, 2023. https://apps.who.int/iris/handle/10665/254610
6. Knoll AD, MacLennan RN. Prevalence and correlates of depression in Canada: Findings from the Canadian Community Health Survey. *Can Psychol Psychol Can*. 2017;58(2):116-123. doi:10.1037/cap0000103
7. Duman RS, Aghajanian GK, Sanacora G, Krystal JH. Synaptic plasticity and depression: new insights from stress and rapid-acting antidepressants. *Nat Med*. 2016;22(3):238-249. doi:10.1038/nm.4050
8. Kim J, Farchione T, Potter A, Chen Q, Temple R. Esketamine for Treatment-Resistant Depression — First FDA-Approved Antidepressant in a New Class. *N Engl J Med*. 2019;381(1):1-4. doi:10.1056/NEJMp1903305
9. McIntyre RS, Filteau MJ, Martin L, et al. Treatment-resistant depression: Definitions, review of the evidence, and algorithmic approach. *J Affect Disord*. 2014;156:1-7. doi:10.1016/j.jad.2013.10.043
10. Bergfeld IO, Mantione M, Figee M, Schuurman PR, Lok A, Denys D. Treatment-resistant depression and suicidality. *J Affect Disord*. 2018;235:362-367. doi:10.1016/j.jad.2018.04.016
11. Johnston KM, Powell LC, Anderson IM, Szabo S, Cline S. The burden of treatment-resistant depression: A systematic review of the economic and quality of life literature. *J Affect*

Disord. 2019;242:195-210. doi:10.1016/j.jad.2018.06.045
12. Interventional Psychiatry Clinical Program 2 | Research at St. Michael's Hospital. Accessed February 8, 2023. https://research.unityhealth.to/research-programs/interventional-psychiatry/interventional-psychiatry-clinical-program2/
13. IV Ketamine | Research at St. Michael's Hospital. Accessed February 8, 2023. https://research.unityhealth.to/iv-ketamine/
14. rTMS | Research at St. Michael's Hospital. Accessed February 8, 2023. https://research.unityhealth.to/rtms-2/
15. Electroconvulsive Therapy | Research at St. Michael's Hospital. Accessed February 8, 2023. https://research.unityhealth.to/electroconvulsive-therapy/
16. Holmes EA, O'Connor RC, Perry VH, et al. Multidisciplinary research priorities for the COVID-19 pandemic: a call for action for mental health science. *Lancet Psychiatry*. 2020;7(6):547-560. doi:10.1016/S2215-0366(20)30168-1
17. Digital Therapeutics | Research at St. Michael's Hospital. Accessed February 8, 2023. https://research.unityhealth.to/digital-therapeutics/
18. Torous J, Kiang MV, Lorme J, Onnela JP. New Tools for New Research in Psychiatry: A Scalable and Customizable Platform to Empower Data Driven Smartphone Research. *JMIR Ment Health*. 2016;3(2):e16. doi:10.2196/mental.5165
19. Vaidyam A, Halamka J, Torous J. Actionable digital phenotyping: a framework for the delivery of just-in-time and longitudinal interventions in clinical healthcare. *mHealth*. 2019;5:25-25. doi:10.21037/mhealth.2019.07.04
20. Henson P, Barnett I, Keshavan M, Torous J. Towards clinically actionable digital phenotyping targets in schizophrenia. *Npj Schizophr*. 2020;6(1):13. doi:10.1038/s41537-020-0100-1
21. Torous J, Jän Myrick K, Rauseo-Ricupero N, Firth J. Digital Mental Health and COVID-19: Using Technology Today to Accelerate the Curve on Access and Quality Tomorrow. *JMIR Ment Health*. 2020;7(3):e18848. doi:10.2196/18848
22. Torous J, Bucci S, Bell IH, et al. The growing field of digital psychiatry: current evidence and the future of apps, social media, chatbots, and virtual reality. *World Psychiatry*. 2021;20(3):318-335. doi:10.1002/wps.20883
23. Murray E, Hekler EB, Andersson G, et al. Evaluating Digital Health Interventions. *Am J Prev Med*. 2016;51(5):843-851. doi:10.1016/j.amepre.2016.06.008
24. Nickels S, Edwards MD, Poole SF, et al. Toward a Mobile Platform for Real-world Digital Measurement of Depression: User-Centered Design, Data Quality, and Behavioral and Clinical Modeling. *JMIR Ment Health*. 2021;8(8):e27589. doi:10.2196/27589
25. Moshe I, Terhorst Y, Opoku Asare K, et al. Predicting Symptoms of Depression and Anxiety Using Smartphone and Wearable Data. *Front Psychiatry*. 2021;12:625247. doi:10.3389/fpsyt.2021.625247
26. Lee S, Kim H, Park MJ, Jeon HJ. Current Advances in Wearable Devices and Their Sensors in Patients With Depression. *Front Psychiatry*. 2021;12:672347. doi:10.3389/fpsyt.2021.672347
27. Katsis CD, Katertsidis NS, Fotiadis DI. An integrated system based on physiological signals for the assessment of affective states in patients with anxiety disorders. *Biomed Signal*

Process Control. 2011;6(3):261-268. doi:10.1016/j.bspc.2010.12.001
28. McClintock SM, Reti IM, Carpenter LL, et al. Consensus Recommendations for the Clinical Application of Repetitive Transcranial Magnetic Stimulation (rTMS) in the Treatment of Depression: (Consensus Statement). *J Clin Psychiatry*. 2018;79(1):35-48. doi:10.4088/JCP.16cs10905
29. Oudejans LCJ, van Velzen M, Dahan A. Ketamine Analgesia. In: *Neuropathology of Drug Addictions and Substance Misuse*. Elsevier; 2016:541-550. doi:10.1016/B978-0-12-800212-4.00050-9
30. Phillips JL, Norris S, Talbot J, et al. Single, Repeated, and Maintenance Ketamine Infusions for Treatment-Resistant Depression: A Randomized Controlled Trial. *Am J Psychiatry*. 2019;176(5):401-409. doi:10.1176/appi.ajp.2018.18070834
31. Hermida AP, Glass OM, Shafi H, McDonald WM. Electroconvulsive Therapy in Depression. *Psychiatr Clin North Am*. 2018;41(3):341-353. doi:10.1016/j.psc.2018.04.001
32. Buckman JEJ, Underwood A, Clarke K, et al. Risk factors for relapse and recurrence of depression in adults and how they operate: A four-phase systematic review and meta-synthesis. *Clin Psychol Rev*. 2018;64:13-38. doi:10.1016/j.cpr.2018.07.005
33. Catan G, Espanha R, Veloso Mendes R, Toren O, Chinitz D. The Impact of eHealth and mHealth on doctor behavior and patient involvement: an Israeli and Portuguese comparative approach. *Stud Health Technol Inform*. 2015;210:813-817.
34. Qudah B, Luetsch K. The influence of mobile health applications on patient - healthcare provider relationships: A systematic, narrative review. *Patient Educ Couns*. 2019;102(6):1080-1089. doi:10.1016/j.pec.2019.01.021
35. Henson P, Wisniewski H, Hollis C, Keshavan M, Torous J. Digital mental health apps and the therapeutic alliance: initial review. *BJPsych Open*. 2019;5(1):e15. doi:10.1192/bjo.2018.86
36. Torous J, Lipschitz J, Ng M, Firth J. Dropout rates in clinical trials of smartphone apps for depressive symptoms: A systematic review and meta-analysis. *J Affect Disord*. 2020;263:413-419. doi:10.1016/j.jad.2019.11.167
37. Hollis C, Falconer CJ, Martin JL, et al. Annual Research Review: Digital health interventions for children and young people with mental health problems - a systematic and meta-review. *J Child Psychol Psychiatry*. 2017;58(4):474-503. doi:10.1111/jcpp.12663
38. REDCap. Accessed February 8, 2023. https://www.project-redcap.org/
39. Oura Ring. Smart Ring for Fitness, Stress, Sleep & Health. Accessed February 8, 2023. https://ouraring.com/
40. Chee NI, Ghorbani S, Golkashani HA, Leong RL, Ong JL, Chee MW. Multi-Night Validation of a Sleep Tracking Ring in Adolescents Compared with a Research Actigraph and Polysomnography. *Nat Sci Sleep*. 2021;Volume 13:177-190. doi:10.2147/NSS.S286070
41. Altini M, Kinnunen H. The Promise of Sleep: A Multi-Sensor Approach for Accurate Sleep Stage Detection Using the Oura Ring. *Sensors*. 2021;21(13):4302. doi:10.3390/s21134302
42. Spitzer RL, Kroenke K, Williams JBW, Löwe B. A Brief Measure for Assessing Generalized Anxiety Disorder: The GAD-7. *Arch Intern Med*. 2006;166(10):1092. doi:10.1001/archinte.166.10.1092
43. Kroenke K, Spitzer RL. The PHQ-9: A New Depression Diagnostic and Severity Measure. *Psychiatr Ann*. 2002;32(9):509-515. doi:10.3928/0048-5713-20020901-06

44. Topp CW, Østergaard SD, Søndergaard S, Bech P. The WHO-5 Well-Being Index: A Systematic Review of the Literature. *Psychother Psychosom*. 2015;84(3):167-176. doi:10.1159/000376585
45. Wenze SJ, Miller IW. Use of ecological momentary assessment in mood disorders research. *Clin Psychol Rev*. 2010;30(6):794-804. doi:10.1016/j.cpr.2010.06.007
46. van Genugten CR, Schuurmans J, Lamers F, et al. Experienced Burden of and Adherence to Smartphone-Based Ecological Momentary Assessment in Persons with Affective Disorders. *J Clin Med*. 2020;9(2):322. doi:10.3390/jcm9020322
47. aan het Rot M, Hogenelst K, Schoevers RA. Mood disorders in everyday life: A systematic review of experience sampling and ecological momentary assessment studies. *Clin Psychol Rev*. 2012;32(6):510-523. doi:10.1016/j.cpr.2012.05.007
48. Colombo D, Fernández-Álvarez J, Patané A, et al. Current State and Future Directions of Technology-Based Ecological Momentary Assessment and Intervention for Major Depressive Disorder: A Systematic Review. *J Clin Med*. 2019;8(4):465. doi:10.3390/jcm8040465
49. Intille S, Haynes C, Maniar D, Ponnada A, Manjourides J. μEMA: Microinteraction-based ecological momentary assessment (EMA) using a smartwatch. In: *Proceedings of the 2016 ACM International Joint Conference on Pervasive and Ubiquitous Computing*. ACM; 2016:1124-1128. doi:10.1145/2971648.2971717
50. Nguyen B, Nigro M, Rueda A, Krishnan S, Bhat V. Digital Phenotype representation by Statistical and Data-Driven approaches with Digital Health Data. *ICASSP 2022 - 2022 IEEE Int Conf Acoust Speech Signal Process ICASSP 2022*. Published online 2022.
51. Barnett I, Torous J, Reeder HT, Baker J, Onnela JP. Determining sample size and length of follow-up for smartphone-based digital phenotyping studies. *J Am Med Inform Assoc*. 2020;27(12):1844-1849. doi:10.1093/jamia/ocaa201
52. Dresing T, Pehl T, Schmieder C. Manual (on) Transcription. Transcription Conventions, Software Guides and Practical Hints for Qualitative Researchers. 3rd English Edition. 2015. Marburg Available Online: http://www.audiotranskription.de/english/transcription-practicalguide.htm. Access: 06.11.2023
53. Clarke V, Braun V. Using thematic analysis in psychology. *Qualitative Research in Psychology*. 2006;*3*(2): 77-101. doi: 10.1191/1478088706qp063oa

10. APPENDICES

Appendix A. Self-Report Questionnaires & Clinical Assessments

Appendix B. CRF – Intake Form

Appendix C. CRF - Eligibility

Appendix D. Email Drafts

Appendix E. Screening & Baseline Visit Script
