# Supplementary material for: Digital health monitoring for adults with treatment-resistant depression: Observational feasibility study protocol
Source: PLoS One. 2025 Oct 24;20(10):e0333484. doi: 10.1371/journal.pone.0333484 (PMC12551856; doi:10.1371/journal.pone.0333484)
Supplement: S4 File — (PDF) [file pone.0333484.s004.pdf]

Letter of Information and Consent to Participate in a Research Study

**Study Title:**	**Determining the Feasibility of Digital Interventions for Adults with Treatment-Resistant Depression**
**Principal Investigator:**	**Dr. Venkat Bhat,** MD MSc FRCPC DABPN<br>Director, Interventional Psychiatry Program<br>St. Michael's Hospital, Unity Health Toronto<br>Assistant Professor, Department of Psychiatry, University of Toronto<br>Phone: (416) 360-4000 x76404
**Co-Investigators:**	**Dr. Perry Menzies,** MD MSc FRCPC<br>Clinician Teacher, St. Michael's Hospital<br>Lecturer in Psychiatry, University of Toronto<br><br>**Dr. Wendy Lou,** PhD<br>Professor, Division Head of Biostatistics<br>Dalla Lana School of Public Health, University of Toronto<br><br>**Dr. Sridhar Krishnan,** PhD<br>Professor, Signal Analysis Research Lab<br>Toronto Metropolitan University
**Study Coordinator:**	**Dr. Karisa Parkington,** PhD<br>Postdoctoral Research Fellow, Interventional Psychiatry Program<br>St. Michael's Hospital, Unity Health Toronto<br>Study Contact Email: IAMGOLD@unityhealth.to
**Study Staff:**	**Fathima Adamsahib,** BSc, PA-C<br>Physician Assistant, Interventional Psychiatry Program<br>St. Michael's Hospital, Unity Health Toronto<br><br>**Reinhard Janssen Aguilar,** MA, MD<br>Postdoctoral Clinical Fellow, Interventional Psychiatry Program<br>St. Michael's Hospital, Unity Health Toronto<br><br>**Gyu Hee (Sarah) Lee**<br>Research Assistant, Interventional Psychiatry Program<br>St. Michael's Hospital, Unity Health Toronto
**Study Funder:**	Miner's Lamp Innovation Fund in Prevention and Early Detection of Severe Mental Illness; Department of Psychiatry, University of Toronto

Conflict of Interest Statement

St. Michael's Hospital is using funding awarded by the Department of Psychiatry at University of Toronto for the "Miner's Lamp Innovation Fund in Prevention and Early Detection of Severe Mental Illness" to conduct this study. The Principal Investigator of this study has no financial or competing interests to declare.

Introduction

You are being asked to consider participating in this research study because you have a diagnosis of Major Depressive Disorder (MDD) classified as Treatment-Resistant Depression (TRD) and are enrolled in the Interventional Psychiatry Program at St. Michael's Hospital to receive clinical treatment.

All research is voluntary – you do not have to participate, and you can withdraw at any time.

Before agreeing to take part in this research study, it is important that you read the information in this informed consent form. It includes details we think you need to know in order to decide if you wish to take part in the study. If you have any questions, ask an investigator or a research team member.

You should be aware that it is possible that the St. Michael's Hospital study investigator might also be your treating doctor.

If you choose to participate in the study, you will need to sign this Letter of Information and Consent form. You should not sign this form until you are sure you understand the information. You may also wish to discuss the study with others, such as your friends, family, and/or family doctor.

Background and Purpose of the Research Study

The purpose of this study is to learn whether two digital platforms are feasible for collecting data related to the mental health of adults receiving treatment for TRD in the Interventional Psychiatry Program at St. Michael's Hospital.

These platforms include an electronic data capture platform (REDCap) and a wearable device (Oura Ring). The REDCap platform allows the clinical team to enter your questionnaire responses (collected as part of your clinical care) into an electronic format for use in research aimed at investigating patients' responses to clinical treatment. The wearable device is a commercially available smart-ring (named "Oura Ring"), which collects data about your activity, sleep and physiological signals. Data collected by the Oura Ring are obtained passively (henceforth *passive data*), as they do not require any input from you to be generated. The Oura Ring is durable, lightweight, and comfortable to wear during daily activities; it has been successfully used in various research studies related to sleep and/or mental health. Both platforms may be useful for monitoring the treatment of people suffering from TRD, which can affect one's thoughts, emotions, and behaviour.

We hope to use regular assessments in the future to help improve our care here in the program, a procedure known as Measurement-Based Care (MBC). Regular assessments allow one to better understand their thoughts and emotions by enabling data visualisation and self-monitoring by the patient; they also help clinicians better visualise how a patient is responding to treatment. When compared to traditional care, MBC improves patient outcomes and the quality of patient care and optimises monitoring of symptom reduction. Additionally, MBC has been shown to have a positive

impact on the therapeutic relationship, enhance the decision-making process and allow for individualised treatment. We will evaluate whether REDCap is feasible to deliver regular assessments as part of clinical care and if these can help us better understand how symptoms change with treatment (over time). We will also be able to detect and compare measures of treatment response, remission, and relapse using these self-report assessments.

Studies have shown that the Oura Ring provides accurate measures of sleep and physiological parameters. Taken together, assessments on REDCap and measures via the Oura Ring may facilitate one's insights into their own health, while also allowing the care team to monitor symptom changes and treatment response.

This study will examine the feasibility of implementing the REDCap platform and Oura Ring alongside neuropsychiatric treatment for TRD in the Interventional Psychiatry Program at St. Michael's Hospital. This will allow us to digitally monitor patient symptoms and measure sleep, activity and physiological signals relevant to mental health monitoring in real time. The findings from this study will help guide future studies in assessing the effectiveness of these platforms for treatment monitoring in a larger number of participants. Once effectiveness is demonstrated, we can expand the use of these digital platforms for use in other mental illnesses and enable real-time measurement-based care, which could enhance clinical decision-making, improve patient outcomes, and prevent illness relapse.

Research Study Design and Duration

Study Design

This is an observational study that will aim to evaluate the feasibility of the REDCap platform and Oura Ring to collect active and passive digital data of relevance to mental health during clinical treatment. The results of this study will help us plan a future study looking at the effectiveness in larger groups of participants. We will perform this study with 200 participants with TRD throughout the duration of their clinical treatment. All participants will have their questionnaire and clinical assessment data entered into REDCap. Depending on availability of wearable devices, interested participants can also opt to use an Oura Ring in combination with the REDCap platform for the duration of the study. We anticipate that approximately 60 Oura Rings will be available for the study.

Participant Population and Study Enrollment

This study will include 200 participants aged 18 years and older with a diagnosis of MDD and TRD who are enrolled at the Interventional Psychiatry Program at St. Michael's Hospital, Unity Health Toronto, for clinical treatment.

Study Duration

Overall, this study will run for about three years. Your involvement in the research study will depend on the duration of your clinical treatment (typically 2 - 4 weeks) and whether you use an Oura Ring (which requires a 2-week baseline period prior to starting treatment).

Description of Research Activities

Research Procedures

Screening and Baseline Visit (30 - 60 minutes)

If you decide to participate in the study, you will be asked to complete a Screening and Baseline Visit. If you are receiving an Oura Ring, this visit will occur in-person at the Interventional Psychiatry Program at St. Michael's Hospital (30 Queen Street, 17CC) approximately 3 weeks before your first treatment session (because a baseline period is needed to ensure accurate data collection and establish baseline parameters). If you are not receiving an Oura Ring (i.e., REDCap only), this visit will take place at your first treatment session.

During the Screening and Baseline Visit, we will confirm your eligibility for this study (based on information collected during IPP intake) and have you complete baseline measures of mental health (as part of clinical care). If you opt to use the Oura Ring, we will provide you with an appropriately sized device and will help you download and set up its mobile application (Oura app) on your smartphone. You will be expected to continue using Oura (if applicable) throughout your clinical treatment.

The standard of care that you receive from your healthcare care practitioner will not be changed if you decide to participate in this study. All research interventions and activities will be in addition to your usual standard of care.

At the beginning of the Screening and Baseline Visit, a member of the study team will review your demographic information and medical history (provided during intake to the Interventional Psychiatry Program) and confirm your eligibility for the study. The demographic information will include the following: (1) age; (2) sex; (3) gender; (4) marital status; (5) ethnicity; (6) level of education; (7) handedness; (8) employment; and (9) personal history (i.e., personality, difficulties in school/work etc.). The medical history will include the following: (1) previous diagnoses; (2) current diagnoses; (3) previous psychological therapies/treatments; (4) previous and current medications (e.g., psychopharmacological treatments, psychoactive medications); (5) other medical history; (6) family psychiatric history; and (7) substance use.

Your responses to questionnaires or assessments completed as part of your clinical care will be entered into REDCap by the clinical team after your treatment session. Your responses will be associated with a de-identified study identification (ID) number; this will ensure your active data remains de-identified and you will not be personally identified on the REDCap platform.

If you are also interested in wearing the Oura Ring for passive data collection (and if a properly fitting device is available), you will wear the device on a daily basis throughout your participation in the study, and will return it at your last treatment session. The smart-ring will continuously collect passive data on your sleep, activity, and physiological signals. You will receive the credentials to a de-identified study email account to access your account on the Oura app. Because you will use a study account to access your Oura account, data collection will occur in a de-identified manner during your participation in the study. This means that you will not be identified on the Oura platform.

Demographic Information and Medical History

Self-Report Assessments (via REDCap)

In order for us to gain a better understanding of the feasibility of using an electronic data capture platform and understand treatment response, your clinical questionnaire/assessment data will be entered into the REDCap platform after each treatment session in the Interventional Psychiatry Program. If you use REDCap in another program or organization, this information will not be linked

to this study. If you have any questions about the REDCap platform, you should contact the Study Coordinator at IAMGOLD@unityhealth.to.

The frequency and duration of the assessments on REDCap will depend on your treatment protocol in the Interventional Psychiatry Program. All participants will complete the Generalized Anxiety Disorder Scale (GAD-7) – seven questions about anxiety symptoms - on each treatment day, as well as a measure of depression symptoms: some participants will complete the Patient Health Questionnaire (PHQ-9) – nine questions about depressive symptoms on REDCap, while others may complete the Montgomery-Asberg Depression Rating Scale (MADRS) interview with their clinical team at the treatment visit. **Passive Data (via Oura Ring)**

If you use the Oura Ring in this study, this device will be used to passively collect your physiological data, such as heart rate, heart rate variability, body temperature, and respiratory rate. This wearable device can also measure activity levels and monitor sleep (e.g., time spent in bed, sleep stages, number of hours of sleep) through its sensors . As mentioned earlier, you will receive de-identified study credentials (dummy email address and password) which you will use to log in and access your Oura app. Although the Oura app asks for your personal information when you first log in (e.g., sex, weight and height), **please DO NOT enter any personal information on the Oura app as data collection is de-identified.**

The implementation of the Oura Ring in the study will help us understand the feasibility of using this device for monitoring a person's activity patterns and behaviour throughout their involvement in this study and in response to clinical treatment. If the implementation of wearable devices such as the Oura Ring successfully allows for the ongoing monitoring of mental health symptoms, their use in psychiatric environments may improve the examination, early detection, and treatment of MDD and, potentially, other mental illnesses in the future. Figure 1 illustrates a person using the Oura Ring and its app.

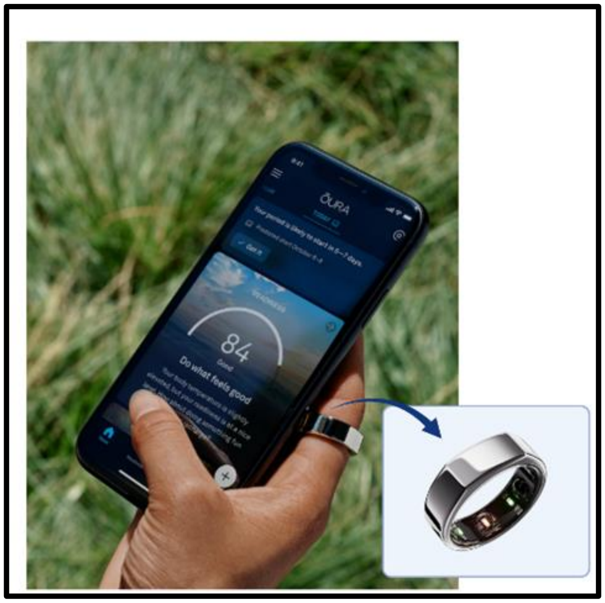

Figure 1. The Oura Ring wearable device and its respective mobile app (designed with images from https://ouraring.com/).

Any information used to register you, entered by you in the Oura portal, or generated by the Oura Ring, will be accessible by Oura. Unity Health Toronto will not give Oura any of your identifiers. Oura may use your de-identified information in the future to improve and market their ring (from which they may profit) indefinitely. De-identified data is handled in accordance with Oura's privacy policy, which can be accessed at https://cloud.ouraring.com/legal/teams/privacy-policy.

You should know that Oura uses tools to monitor your activity on their websites. These tools will identify you by IP address, and will track which websites you visit and some of your activity on the website (such as how long you stay on the site). Oura may, at any time in the future, choose to use tools that also track and collect the information that you enter into a website, or that is collected by the ring and visible to you in their app; this may include data generated in this study.

The parameters to be collected by the Oura Ring are listed as follows:

- Resting heart rate
- Heart rate variability (HRV)
- Respiratory rate
- Daily activity (e.g., distance covered, calories)
- Blood oxygen saturation
- Body temperature variation (delta)
- Sleep metrics (e.g., sleep staging, total sleep time, latency)
- Individual's readiness, sleep, and activity scores
- Workout heart rate

It is important to note that the Oura Ring collects data such as heart rate and daily movement, which may be sensitive to certain private activities (i.e., moments of intimacy) that you may not want to share. If a moment of intimacy were to happen, the data may show an elevated heart rate, but we have no way of inferring the exact activity that you are doing, only that you are being active. If you feel uncomfortable wearing the ring during these moments, please take it off and put it back on when finished.

Two-Zone Approach

Data collected via REDCap and the Oura Ring will be de-identified as participants' accounts will be associated with a de-identified study ID and dummy email address with password. Data collected via REDCap will be de-identified because the de-identified email addresses will be entered into the participant list; emails containing survey links will be forwarded to participants' personal email addresses (in the same manner as Oura emails). During data analysis, de-identified data will be provided to the analysts. This will be achieved using a two-zone approach. The two-zone approach divides study team members into two groups; identified and de-identified. As the names suggest, the identified group will have access to identified (i.e. demographic data) and de-identified information; personally identifying information (name and email address) will be recorded in a password-protected master file only accessible to authorized research personnel. Alternatively, the de-identified group will only have access to the de-identified information (responses to questionnaires and Oura data). This will ensure that the data analysts of the team do not have access to any of your identifiers to protect your privacy.

Clinical Care

Upon confirming your eligibility, you will be able to begin your clinical treatment. Importantly, participants with an Oura Ring will have a 2-week period between the first study visit and the beginning of their clinical treatment for baseline parameter establishment by the Oura Ring.

Participants without an Oura Ring will complete the Screening and Baseline Visit on the first day of treatment and can begin receiving clinical treatment immediately. You will continue to receive clinical treatment as per the standard of care throughout this study. During each clinical visit, you will complete self-report questionnaires and/or clinician-administered assessments as part of your standard of care. For the purposes of this study, we are only interested in using your scores on the GAD-7, PHQ-9, and/or MADRS in the REDCap platform, not the C-SSRS. The study observations will not interfere with the care you receive at the Interventional Psychiatry Program.

Summary

As a participant in this study, you are providing consent for the clinical team to enter your responses to self-report questionnaires (GAD-7, PHQ-9) and/or a clinician-administered assessment (MADRS) measuring anxiety and depression
in addition to your standard clinical care for TRD in the Interventional Psychiatry Program.

Based on device availability, you may also have the option to wear a smart-device (Oura Ring) daily for the duration of the study (2-week baseline + duration of treatment) to passively collect activity, sleep, and physiological measures (based on device availability).

You will be asked to return the device at the end of your last treatment appointment.

The clinical team will schedule and coordinate your visits with the Interventional Psychiatry Program clinic process.

Participant responsibilities

It is important to remember the following if you choose to participate in this study:

- Ask your study team about anything that worries you.
- Tell study staff about any changes in your health.
- Tell study staff if your depression becomes worse.
- Tell study staff if you are having thoughts about hurting yourself or anyone else.
- Tell your study team if you change your mind about being in this study.
- Tell your study team if you are considering enrolling in another study.
- Complete the questionnaires and/or clinician-administered assessments included as part of your standard of care each treatment day*
- Wear the Oura Ring on a daily basis (if applicable) and sync data to your smartphone at least once a day**

If you have any questions about the digital platforms (REDCap and/or Oura) or need help troubleshooting technical issues, please contact the research team via email at IAMGOLD@unityhealth.to; this email is checked regularly by the Study Coordinator and research team. You can also contact the Study Coordinator directly by email karisa.parkington@unityhealth.to.

*A member of the clinical team will enter your responses into REDCap at the end of each treatment visit, for use in research.

**A member of the research team will monitor wearable data compliance on a weekly basis. If your Oura data is missing for 3 or more days over the past 7 days, the research team will notify

your clinical care team who will discuss with you further. You may be referred to the research team to troubleshoot any technical issues.

Potential Risks of Study Intervention

REDCap Platform

Self-report assessments on the web-based platform (REDCap) contain questions regarding sensitive personal information (mental health symptoms). You may experience a negative reaction or feelings of distress when responding to some of the questions. If this happens, please notify the clinical team so your clinical care can be accommodated accordingly; your participation in the REDCap components of the study may be paused or stopped or you can also choose to withdraw from the study. Participation in this study is completely voluntary and you may withdraw at any time. To inform digital intervention feasibility for future studies, you may be contacted by the study team to understand reasons for pausing or withdrawing data collection via REDCap.

Most importantly, if you need immediate assistance, please call 911 or go to the closest Emergency Room.

The Study Coordinator and research team are available to answer any questions you might have regarding the study or the technology platforms (IAMGOLD@unityhealth.to).

Oura Ring Wearable

If you agree to participate, you may be asked to use the Oura Ring wearable device (if available) and its app throughout your involvement in the study. You may experience some burden or a negative reaction when using the wearable device and/or its app as you will be asked to wear the device whenever possible and to recharge it when necessary (approximately every 3-4 days). You may also experience negative feelings or reactions when accessing your data on the wearable app. If any of these happen, you can pause or stop using the wearable device and wearable app, or you can also choose to withdraw from the study. Additionally, you may have an allergic reaction while using the wearable device; in this case, you should stop using the device and contact your family physician. Most importantly, if you experience a negative reaction or distress, you may contact the study coordinator, as they are available to answer any questions you might have regarding the study, the wearable and its app, or where to seek assistance if needed. If you need immediate assistance, please call 911 and go to the closest Emergency Room.

De-Identified Data Security

There is also a very small possibility that your data could be stolen; however, the REDCap platform, the Oura app, and the data storage systems have many features to keep your data secure. In the rare event that this were to happen, someone would know the information from your accounts, such as data you reported on the surveys or information from your internet use records, but they would not have access to personally-identifying information and would not be able to tie the de-identified data back to you. No personal information will be collected through REDCap or the Oura platform. All digital data (REDCap assessments, demographic information, and medical history; or passive data collected by Oura) will only be associated with a de-identified study ID number and dummy email address; personal information (name and personal email address) will be collected separately (in a password-protected master document available only to authorized research personnel) and stored within a secure electronic database maintained by Unity Health Toronto (Citrix Network folder).

Potential Benefits

Monitoring your own physical and mental health data can have a positive impact on your health lifestyle and can help promote healthy behaviours. There are also potential benefits to society as this study may help examine mechanisms and interventions to support vulnerable groups to promote better mental health outcomes, reduce maladaptive coping behaviors, and increase adherence to using web-based platforms and wearables, alongside evidence-based recommendations. The information collected on mental health symptoms (anxiety, depression, sleep) and physiological markers (such as heart rate and heart rate variability, body temperature, respiration rate, and blood oxygenation) will help us gain additional insights into MDD, TRD, and response to treatment. Therefore, your participation may help others in the future as a result of the knowledge gained from the research. However, it is not possible to predict whether you will benefit directly from participation in this study.

Alternatives to Participation

You do not have to join this study to receive treatment for your condition. If you decline to be in this study, your care will not be affected.

In making your decision, you should keep in mind that being in a study is not a form of treatment and that participating in a study is not the same as being treated. Participation in this research study does not replace routine physical examinations or visits to your regular doctor. We recommend that you discuss these and other options with the investigator and your family doctor so that you can make a well-informed decision about participating in this study.

Privacy and Confidentiality of Your Personally Identifiable Information and Study Data

This section describes how your identifiable information and study data will be accessed, disclosed, and stored during this study. All persons involved in the study are committed to respecting your privacy. Other than the individuals or groups described in this section, no persons will have access to your identifiable information without your consent, unless required by law.

Personally identifying information is any information that could be used to identify you and includes your name, address, date of birth, or health card number.

Clinical data is information that is collected as routine part of clinical care in the Interventional Psychiatry Program at St. Michael's Hospital. In this case, clinical data refers to the assessments completed as part of the treatment process (GAD-7, PHQ-9 or MADRS).

Study data is information that is generated by and/or collected for a study, which includes the active data entered into the REDCap platform and the passive data collected by the Oura Ring and app.

Protecting Your Privacy

The study personnel will make every effort to keep your personally identifying information private and confidential in accordance with all applicable privacy legislation, including the Personal Health Information Protection Act (PHIPA) of Ontario.

In addition to the study team, other authorized employees of Unity Health Toronto may have access to your personally identifying information so that they can carry out regulatory or institutionally required duties. Unity Health Toronto may also store personally identifying information that is

collected or used for these duties for a period of time in accordance with regulations and institutional policies.

The study doctor at St. Michael's Hospital is in control of the key that links your study number to your personal information and will keep it stored separately from the study data. No personally-identifying information will be allowed off-site in any form unless required by law or as described in this consent form.

Web-based and Wearable Platforms

No personal identifiable data will be collected through the web-based survey platform (REDCap) or the wearable device (Oura Ring). All data will be de-identified using the de-identified study ID number and dummy email address. That means that we do not want to identify your data as belonging to you. **You should never provide your name or any other piece of information that identifies you through the Oura app, even if requested by this platform**. The demographic information (e.g., age, gender) and medical history (e.g., previous diagnoses, medication history) asked for during the intake process and entered into REDCap will be collected and stored separately from the Oura Ring data. This demographic data will only be linked to the active data on REDCap via a de-identified study ID number and will not be linked with the wearable data until the final analysis of the data.

Data collected by the Oura Ring device and its app will be securely stored on the Oura's respective servers; this allows your data to be visualized on your smartphone app. However, you will remain de-identified on the Oura servers, as your data will be collected using a de-identified study email. After the study is completed, the research team will request the wearable company to delete all your data from their servers and it will be transferred to an external encrypted and password-protected hard-drive.

Use of Email for Research

There are common risks of using email messaging to communicate:

- Information travels electronically and is not secure in the way a phone call or regular mail would be.
- If someone sees these emails, they may know that you are a participant in this study or see the health information included in the email.
- Emails may be read or saved by your internet or phone provider (e.g., Rogers, your workplace, "free internet" providers).
- Copies of an email may continue to exist, even after efforts to delete the email have been made.
- There is always a chance with any unencrypted email, however remote, that it could be intercepted or manipulated.

Do not use email and/or text messaging for medical emergencies. If you require immediate help, call your clinic or care provider, or seek emergency services.

Personally-Identifying Information and Study Data Storage and Retention

All personally identifying data used in this study will be securely stored. All the study data will be collected by a secure electronic software transferred to our servers at Unity Health Toronto.

Your research records will be kept for 7 years after study completion at St. Michael's Hospital in a highly secure and confidential manner. Active data will be entered and stored on the REDCap server whereas passive data collected by the wearable device will be stored on the Oura servers and later securely transferred to an external encrypted and password-protected hard-drive. All study data may be used for other research or analyses by the investigators, or by other researchers. Future studies where this data will be used or shared will be subject to the approval of the Research Ethics Board (REB).

Personally identifying information collected for research purposes will be kept by the Principal Investigator and Unity Health Toronto for as long as required by Unity Health Toronto's policy (currently 7 years after this study ends), at which point any documents with personally identifying information will be destroyed.

Study Registration and Results

Registration

A description of this observational feasibility trial will be available on http://www.ClinicalTrials.gov. This website will not include information that can identify you. At most, the website will include a summary of the results. You can search this website at any time.

The registration number for this study is NCT06732089.

Results

The protocol and results of this study may be presented at scientific conferences or published in scientific journals. If you are interested in obtaining the results of the study, you can contact the investigators or research team. We estimate that the results of the study will be available in five years.

Some scientific journals may require us to make the study data available to the journal, its reviewers, or to other researchers. You will never be personally identified in any publication, report, or presentation that may come from this study.

Participation and Withdrawal

Voluntary Participation

Your participation in this study is voluntary. You may decide not to be in this study or to be in the study now and then change your mind later. You may leave the study at any time. If you choose not to participate, there will be no impact to the medical care received at, employment at, or other relationship with Unity Health Toronto now or in the future for you or your family.

Withdrawal from the Study

You may withdraw from this study at any time without any effect on the medical care, employment or other relationship you or your family have at or with Unity Health Toronto. To determine feasibility for future clinical trials, you may be asked why you chose to withdraw from the study; it is up to you to decide if you want to disclose this information or not. You may withdraw from the study at any time without giving a reason. If you are using an Oura Ring for this study, the research team will contact you to schedule a visit to return your Oura Ring.

Your participation in the study may be stopped without your consent for the following reasons:

- The research team decides to stop early
- The research ethics board withdraws permission for this study to continue
- If it is discovered that you do not meet the eligibility requirements

Also, your participation might be discontinued if:

- You fail to adhere to the study procedure.
- You fail to adhere to the clinical treatment at the Interventional Psychiatry Program
- You meet any exclusion criteria (either newly developed or not previously recognized) for the clinical treatment you are receiving at the Interventional Psychiatry Program.

This study may be terminated by the study investigators or by the study sponsor at any time for any reason.

If you are withdrawn from this study or if this study ends early, a study team member will discuss possible next steps with you.

Continued Collection and Use of Your Data after Withdrawal

If you withdraw from the study, any data collected about you up to that time will still be used for analysis. No further data about you will be collected unless it is necessary to follow up on an adverse event that is not resolved at the time of your withdrawal.

We may be required to retain the personally identifying information and study data that we have already collected until after the end of this study (described in the Privacy and Confidentiality section).

You will not have access to the wearable device (Oura Ring) and no other data will be entered into your REDCap profile after your involvement in the study.

Costs and Reimbursement

There are no costs to you for participation in this study.

Compensation for Injury

If you are injured because of your participation in this study, medical care will be provided to you in the same manner as you would ordinarily obtain any other medical treatment. In no way does signing this form waive your legal rights nor release the study doctor(s), sponsor, or involved institution(s) from their legal and professional responsibilities.

Rights as a Participant

If you are harmed as a direct result of taking part in this study, all necessary medical treatment will be made available to you at no cost. By signing this form, you do not give up any of your legal rights against the investigators, sponsor, or involved institutions for compensation, nor does this form relieve the investigators, sponsor, or involved institutions of their legal and professional responsibilities.

New Information About the Research Study

During the study, we may make changes to the study. We may also learn new things about the study that you may need to know. Some of the new information or changes might affect your decision to take part in the study. If so, you will be notified about the new or changed information in a timely manner and we will ask you if you consent to remain in the study. You may be asked to sign a new consent form at that time.

Research Ethics Board Contact

If you have any questions regarding your rights as a research participant, you may contact the Unity Health Toronto Research Ethics Board Office at 416-864-6060 ext. 42557 during business hours (9:00 am to 5:00 pm). Unity Health Toronto is a health network that includes Providence Healthcare, St. Joseph's Health Centre, and St. Michael's Hospital.

The Unity Health Toronto Research Ethics Board is made up of a group of scientists, medical staff, and individuals from other backgrounds (including law and ethics) as well as members from the community. The Board is established by Unity Health Toronto to review studies for their scientific and ethical merit. The Board pays special attention to the potential risks and benefits to the research participant, as well as the potential benefit to society.

Study Contacts

If at any time during this study you have questions about the study or the research activities, you should contact the Principal Investigator, **Dr. Venkat Bhat** at (416) 360-4000 x76404. For technical support and troubleshooting, please contact IAMGOLD@unityhealth.to - this email is checked frequently by the research team.

If you have any questions or concerns outside of the study hours of availability, or would like to speak to the study team for any reason, you can contact **Dr. Venkat Bhat (Principal Investigator)** at venkat.bhat@unityhealth.to, **Dr. Karisa Parkington (Study Coordinator)** at karisa.parkington@unityhealth.to, Dr. Reinhard Janssen Aguilar (Clinical Research Fellow) at reinhard.janssen@unityhealth.to, or **Ms. Sarah Lee (Research Assistant)** at GyuHee.Lee@unityhealth.to.

In case of emergency, please go to the nearest emergency department or call 911 for assistance. Let them know that you are in a study, and the Principal Investigator's name.

Please retain a signed copy of this consent form for your records.

Signature Page: Documentation of Informed Consent

Study Title: Determining the Feasibility of Digital Interventions for Adults with Treatment-Resistant Depression

<u>Participant Statement of Consent</u>

By signing this consent form, I acknowledge that:

- This research study has been sufficiently explained to me both verbally (prior to this visit) and in person, and my questions have been answered to my satisfaction.
- I have been informed of the alternatives to participation in this study.
- I know that I have the right not to participate and the right to withdraw from this study without affecting the quality of medical care at Unity Health Toronto for me and for other members of my family.
- The potential risks and benefits (if any) of participating in this research study have been explained to me.
- I have been told that I have not waived my legal rights nor released the investigator, sponsor, or involved institutions from their legal and professional responsibilities.
- I know that I may ask, now or in the future, any questions I have about this study.
- I have been told that records relating to me and my care will be kept confidential and that no personal information will be disclosed without my permission unless required by law.
- I have been given sufficient time to read the information in this consent form.
- I will be given a signed and dated copy of this consent form.

I consent to participate in this study.

Participant name (print)		Participant/Substitute decision-maker signature		Date	

I have explained to the above-named participant the nature and purpose, the potential benefits, and possible risks of participation in this research study. All questions that have been raised about this study have been answered.

Name of person obtaining consent (print)		Signature of person obtaining consent		Date	
